# Proteolytic enzyme models as tunable preclinical platforms for investigating intervertebral disc degeneration

**DOI:** 10.3389/fcell.2025.1683282

**Published:** 2026-01-12

**Authors:** Jan Gewiess, Annamarie D’Intino, Alejandra Santos, Mauro Alini, Andrea J. Vernengo

**Affiliations:** 1 Department of Orthopaedic Surgery and Traumatology Inselspital, Bern University Hospital, University of Bern, Bern, Switzerland; 2 AO Research Institute Davos, Davos, Switzerland; 3 Henry M. Rowan College of Engineering, Department of Chemical Engineering, Rowan University, Glassboro, NJ, United States

**Keywords:** ChABC, chemonucleolysis, chondroitinase ABC, chymopapain, collagenase, intervertebral disc degeneration, papain, trypsin

## Abstract

Lower back pain (LBP) caused by intervertebral disc (IVD) degeneration is a major global health burden, with significant socioeconomic costs. This review examines proteolytic enzyme-based models for inducing IVD degeneration, focusing on their advantages over mechanical and puncture methods, which often fail to replicate the chronic, multifactorial nature of human degeneration. Enzymatic models, such as chemonucleolysis using chondroitinase ABC (ChABC), chymopapain, collagenase, papain, and trypsin, selectively degrade extracellular matrix components like aggrecan and collagen, mimicking the biochemical and structural changes seen in human IVD degeneration. These models offer controlled, reproducible, and physiologically relevant platforms for studying disease progression and evaluating regenerative therapies. Key findings include the dose- and time-dependent effects of enzymes on disc height loss, biomechanical properties, and matrix composition, as well as their ability to induce mild to moderate degeneration without acute trauma. Comparative studies highlight ChABC’s suitability for early-stage degeneration, while chymopapain and papain produce more severe changes. Enzyme models also provide insights into cellular responses, such as cytokine upregulation and matrix remodeling, which are critical for developing targeted treatments. By enabling precise modulation of degenerative severity, these models hold promise for advancing preclinical research and optimizing regenerative strategies for IVD repair. Looking forward, integrating behavioral and molecular pain outcomes into enzyme-based systems may further enhance their translational value, allowing future models to capture both structural and symptomatic dimensions of disc disease.

## 1 Introduction

Lower back pain (LBP) is a prevalent chronic condition among aging adults, significantly limiting their everyday mobility (Bressler et al., 1999; Hicks et al., 2008; Hoy et al., 2012; Reid et al., 2003). In 2021, low back pain affected an estimated 628.8 million people worldwide, with 266.9 million new cases that year alone, making it one of the most prevalent and disabling musculoskeletal conditions globally (Li et al., 2024). Recent Global Burden of Disease analyses continue to identify LBP as the leading cause of years lived with disability, and its overall burden is projected to keep rising in the coming decades (World Health Organization, 2015; Chang et al., 2024). Several studies have shown a link between LBP and intervertebral disc degeneration (IVD) (Lyu et al., 2021; Diwan and Melrose, 2023; Mohd Isa et al., 2022; Li et al., 2022; Arnbak et al., 2016a; Middendorp et al., 2017; Arnbak et al., 2016b). Given the substantial clinical and economic burden of LBP and its strong association with disc degeneration, there is a critical need for reliable preclinical models that can accurately reproduce key features of human IVD pathology to support the development of effective therapies.

The human spinal column comprises a total of 23 intervertebral discs (IVDs), which play a pivotal role in facilitating spinal mobility and flexibility (Kirnaz et al., 2022). Each IVD comprises three integral components: the central nucleus pulposus (NP), a gelatinous substance with high hydration levels; the annulus fibrosus (AF), a multi-layered structure surrounding the NP; and the cranial and caudal cartilaginous endplates (EP) (Raj, 2008). The NP primarily consists of type II collagen and proteoglycans, with type II collagen loosely arranged across its gel-like matrix (Stergar et al., 2019). The predominant proteoglycan within the NP is aggrecan, distinguished by its elevated glycosaminoglycan (GAG) concentration (Kirnaz et al., 2022). The negatively charged GAGs function to attract and retain water molecules within the NP, resulting in a substantial swelling pressure (Iatridis et al., 1996). This heightened swelling pressure, in conjunction with the compressive strength imparted by type II collagen, equips the NP with the capability to withstand considerable compressive stresses (Kepler et al., 2013).

The cellular composition of the IVD varies by region, with NP cells exhibiting a chondrocyte-like phenotype and elevated expression of proteoglycans (e.g., aggrecan) and type II collagen, critical for ECM maintenance (Kepler et al., 2013; Bron et al., 2009). AF cells range from rounded forms in the inner AF to fibroblast-like in the outer AF (Bron et al., 2009), while EP cells support nutrient exchange (Kim et al., 2009; Malandrino et al., 2014)

IVD degeneration, a multifactorial process often linked to aging, is exacerbated by genetics, obesity, mechanical stress, and smoking (Kirnaz et al., 2022). ECM degradation in the NP, particularly aggrecan and type II collagen, reduces hydration and elasticity due to GAG loss (Risbud and Shapiro, 2014), impairing load-bearing capacity (Kokubo et al., 2008). The AF shifts from a type I to type II collagen-rich matrix, leading to structural disorganization, disc height loss, and increased herniation risk under load (Kokubo et al., 2008; Singh et al., 2009).

The IVD is regarded as the largest avascular structure in the human body, which poses significant challenges to its maintenance and repair. The primary mechanism for nutrient supply to the IVD is microvascular diffusion through the EPs, a process critical for sustaining the disc’s cellular activity and matrix synthesis (De Geer, 2018; Urban et al., 2004). Consequently, the regenerative capacity of the IVD is considered minimal, as the limited nutrient availability restricts the ability of resident cells to repair damage or regenerate the ECM, exacerbating the progression of degeneration (Urban et al., 2004).

Scientists have strived to gain deeper insights into the pathogenesis of IVD degeneration by emulating it *in vivo* and *ex vivo* models. Large animal models have been extensively utilized for *in vivo* studies due to their similarities in loading, geometry, and mechanical and biomechanical properties (Alini et al., 2008). However, they come with drawbacks such as high cost and ethical disadvantages (Vernengo et al., 2023). *Ex vivo* models offer a middle ground between *in vitro* and *in vivo* studies, providing cost-effective and ethically more acceptable alternatives (Vernengo et al., 2023).

In both in in vivo and *ex vivo* systems, common approaches for inducing IVD degeneration include the application of supraphysiological mechanical loads and the use of puncture injuries created with needles or blades through the AF. While these methods are widely used, each presents distinct limitations. Supraphysiological loading often leads to widespread mechanical damage across the disc structure (Kroeber et al., 2002). Needle puncture models tend to produce more localized effects, eliciting acute cellular responses such as viability loss (Fu et al., 2021), decreased disc height, loss of water content, and matrix disorganization (Issy et al., 2013). However, these changes often resemble acute trauma rather than the gradual, progressive nature of human disc degeneration. Human IVD degeneration develops slowly over many years and is driven by several factors—such as the gradual loss of proteoglycans, changes in collagen structure, ongoing inflammation, and reduced nutrient transport (Kirnaz et al., 2022; Oichi et al., 2020; Le Maitre et al., 2005). Mechanical overload and puncture models reproduce only a small portion of the complex, multifactorial nature of human degeneration. This gap has created increasing interest in alternative approaches that can better replicate the controlled, matrix-driven cascade characteristic of naturally occurring degeneration.

Chemonucleolysis, a technique involving the intradiscal injection of proteolytic enzymes, has emerged as a controlled and reproducible method for modeling IVD degeneration. This approach typically employs a fine-gauge needle to deliver enzymes directly into the NP (Seguin et al., 2006; Antoniou et al., 2006), thereby minimizing mechanical disruption to the AF. Once administered, these enzymes degrade critical ECM components, such as aggrecan and collagen, leading to a reduction in hydration, swelling pressure, and load-bearing capacity within the NP. The resulting mechanical imbalance between the NP and AF compromises the disc’s ability to withstand physiological loading and initiates a sequence of degenerative changes that extend across disc compartments. This progression mimics the structural and biochemical features of human IVD degeneration, but on an accelerated timescale. Importantly, enzyme-based models allow investigators to tune degeneration through dose, enzyme type, and timing, which has made them central to contemporary preclinical disc research.

Chemonucleolysis has emerged as a widely used approach for inducing controlled degeneration in preclinical disc research. Despite its broad adoption, the field lacks a focused analysis of how enzyme choice, dose, and implementation influence degeneration outcomes in large-animal models. This review fills that gap by consolidating available studies and highlighting the major considerations that distinguish and guide the use of proteolytic enzyme–based models.

## 2 Review methodology

This review was conducted to synthesize current knowledge on proteolytic enzyme-based models of intervertebral disc (IVD) degeneration, with particular emphasis on their mechanisms of action, comparative properties, and applications in preclinical research. A structured literature search was performed using Google Scholar and PubMed to identify relevant peer-reviewed studies published up to November 2025. Search terms included combinations of “chemonucleolysis,” “chondroitinase ABC,” “papain,” “collagenase,” “chymopapain,” “intervertebral disc degeneration,” “enzyme model,” “*ex vivo*,” and “*in vivo*.” Studies were included if they utilized enzyme injections to induce intervertebral disc (IVD) degeneration in what is defined as large animal models [pig, sheep, goat, dog, and cow (Lee et al., 2021)] in either *in vivo* or *ex vivo* settings, provided cellular, biochemical, histological, or biomechanical outcomes with therapeutic implications, and were published in English in peer-reviewed journals.

**FIGURE 1:**
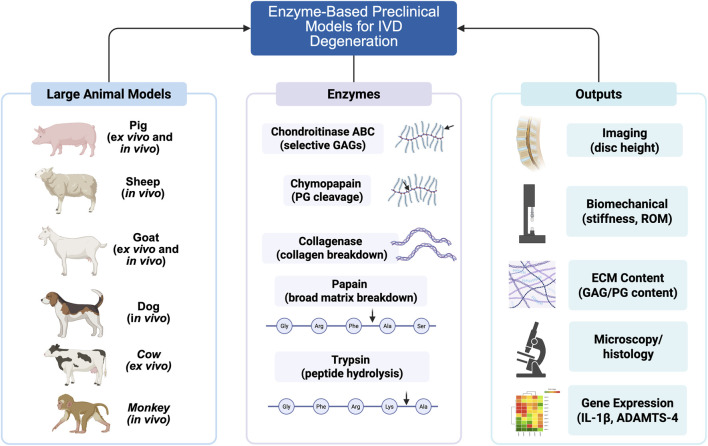
Schematic overview of enzyme-based preclinical models for intervertebral disc (IVD) degeneration. It links large animal models (pig, sheep, goat, dog, cow, monkey) to enzymes—Chondroitinase ABC for selective glycosaminoglycan (GAG) degradation, Chymopapain for proteoglycan (PG) cleavage, Collagenase for collagen breakdown, Papain for broad proteolysis, and Trypsin for peptide hydrolysis. Evaluated outputs include imaging of disc height, biomechanical properties, extracellular matrix (ECM) content, microscopy/histology, and gene expression with IL-1β and ADAMTS-4, reflecting dose- and time-dependent effects. Created in https://BioRender.com.

Studies focused exclusively on surgical techniques, non-enzyme degeneration models (unless used for direct comparison), or non-peer-reviewed sources (e.g., preprints, conference papers, theses, technical reports, patents, books) were excluded. Google Scholar was initially used to maximize coverage, but its limitation in missing some peer-reviewed studies was addressed by supplementing with a PubMed search. Titles were manually scanned to identify potentially relevant studies, duplicates were removed, and full texts were retrieved and reviewed to confirm eligibility. Due to the large volume of Google Scholar results (10,600 records), intermediate exclusion numbers (e.g., non-peer-reviewed sources, content-based exclusions) were not quantified; instead, the process focused on selecting the most relevant studies through iterative screening and full-text review. Key data extracted from each study included enzyme type, delivery method, animal species, target tissue, dose, duration, matrix and cellular responses, and implications for regenerative strategies. Information was organized by enzyme class to facilitate comparison of degenerative profiles and model fidelity across studies (see Figure 1 for a schematic overview).

## 3 Enzyme models

### 3.1 Chondroitinase

Seventeen large-animal studies using chondroitinase to induce IVD degeneration were identified through our literature search, and references for all studies are provided in Tables 1–5. In addition to these seventeen primary studies, we included one additional article (Lü et al., 1997) identified through the reference lists of those papers. Two further publications evaluated therapeutic interventions using the ChABC-induced degeneration model—specifically, mesenchymal stem cell (MSC) transplantation (Ghosh et al., 2012) and hydrogel-conjugated bone morphogenetic proteins (BMPs) (Peeters et al., 2015)—and were therefore included. Across this body of work, degeneration severity was consistently assessed using a common set of outcome measures, including radiographic disc height index, MRI-based quantitative T2/T1ρ relaxation and semi-quantitative grading scales, motion-segment biomechanics, histological grading, and biochemical or spectrophotometric assays of proteoglycan and collagen content (Tables 2–5).

**TABLE 1:** Animal species, spinal sites and application in studies evaluating the degenerative potential of ChABC on large animal IVD.

Animal	Sub-breed	Study	n IVD	Region	Specimen age	Application	Additional features	Follow-up	Needle size (guage)	Volume (mL)	Amount
Dogs	Non-chondroodystrophoid	Fry et al. (1991)	36	L2-7	1-5y	Injection, *in vivo*	No	21d	28	0.05–0.1	10, 50, 100 U/mL
Dogs	Beagle	Lü et al. (1997)	32	L1-7	1y	Injection, *in vivo*	No	7d	31	0.02	25U; 5U
Dogs	Beagle	Ono et al. (1998)	10	L1-7	9–10 m	Injection, *in vivo*	No	52w	23	0.05	250 U/mL
Goats	Dutch white milk goat	Castro et al. (2014)	n.s	Lumbar	3-4y	Injection, *ex vivo*	Physiological loading (sinusoidal load 1 Hz of 150 N average; 100 N amplitude for 16 h; sinusoidal load 1 Hz of 50 N average; 10 N amplitude for 8 h	22d	n.s.	0.1	0.1 U
Goats	Dutch white milk goat	Detiger et al. (2013)	24	L1-6	3-4y	Injection, *in vivo*	No	12w	29	0.13	0.032 U
Goats	Dutch white milk goat	Emanuel et al. (2018)	7	Lumbar	4y	Injection, *in vivo*	No	12w	29	n.s.	0.25 U
Goats	Large-frame	Gullbrand et al. (2017)	20	L1-5	3y	Injection, *in vivo*	No	12w	22	0.2	0.1, 1, 5U
Goats	Large-frame	Gullbrand et al. (2024)	24	C2-C5	2-5y	Injection, *in vivo*	No	12w	22	0.2	2, 5U
Goats	Dutch white milk goat, (Study 1)	Hoogendoorn et al. (2007)	34	Lumbar	3.5y	Injection, *in vivo*	No	26w	29	0.11–0.2	0.25 U/mL
Goats	Dutch white milk goat, (Study 2)	Hoogendoorn et al. (2007)	30	Lumbar	3.5y	Injection, *in vivo*	No	12w	29	0.11–0.2	0.2, 0.25, 0.3, 0.35 U/mL
Goats	Dutch white milk goat	Hoogendoorn et al. (2008)	36	L1-6	Skelettaly Mature	Injection, *in vivo*	No	26w	29	0.14	0.25 U/mL
Goats	Dutch white milk goat	Paul et al. (2018b)	8	L2-5	3-5y	Injection, *ex vivo*	Physiological loading (sinusoidal load 1 Hz at 0.09–0.11 Mpa and 0.1–0.6 Mpa for 16 h per day; 0.09–0.11 Mpa; 1 Hz for 8 h per day)	20d	32	0.1	0.5 U/mL
Goats	Dutch white milk goat	Peeters et al. (2015)	21	L1-6	4y	Injection, *in vivo*	No	24w	29	n.s.	0.25 U/mL
Goats	Large-frame’, only males	Zhang et al. (2020)	12	L1-5	3y	Injection, *in vivo*	No	12w	22	0.2	0.1, 1, 5U
Monkeys	Rhesus monkey	Sugimura et al. (1996)	40	L1-7	Mature	Injection, *in vivo*	No	28w	31	0.02	4U
Monkeys	Cynomolgus	Muramatsu et al. (2020)	43	L4-5	3-5y	Injection, *in vivo*	No	26w	31	0.01	0.25, 10U
Sheep	Merino	Ghosh et al. (2012)	24	L3-6	2y	Injection, *in vivo*	No	3,6 m	29	n.s.	1U
Sheep	Merino	Sasaki et al. (2001)	60	T13-6	5-8y	Injection, *in vivo*	No	4w	21	0.2	1, 5, 50U
Sheep	*Ovis Aries*	Borem et al. (2021)	45	Lumbar	3y	Injection, *in vivo*	No	17w	29	0.2	1U
Bovine	Not specified	Vernengo et al. (2023)	4	Coccygeal	<2y	Injection, *ex vivo*	physiological loading (0.02–0.2 MPa, 0.2 Hz, 2 h)	7d	29	0.1	5U/mL

**TABLE 2:** Imaging findings in studies evaluating the degenerative potential of ChABC on large animal IVD.

Animal species	Study	Follow-up (Days)	DH assessment	DH change	MRI signal assessment	Score change
Dogs	Fry et al. (1991)	5, 7, 14, 21	Dichotomous: Narrowing vs. No Narrowing	Narrowing in 10 U/mL 5d: 66% 7d: 50% 14d: 66% 21d: 83% 50 U/mL 5d: 66% Day 7–21: 100% 100 U/mL Day 5–7: 83% Day 14–21: 100%	Not performed	Not performed
Dogs	Takaishi et al. (1997)	7	Disc height change pre-injection vs. follow up (distance between EP/inferior EP)	−12.8% to −14%	Not performed	Not performed
Dogs	Ono et al. (1998)	7–364	Disc space index ratio ChABC:uninjected (distance between EP/inferior EP width)	7d: −12% 56d: −29.5% 364d: 0%	DeCandido’s grading	DeCandido: 7d: Grade 1 14–56: Grade 3 364d: Grade 2
Sheep	Sasaki et al. (2001)	7–28	DHI pre-injection vs. post injection ((a-c) + ((b-d)) X 100/2 (a-b)	1U 7d: −8.3% 28d: −18.5% 5U 7d: −14.7% 28d: −31.2% 50U 7d: −5.9% 28d: −13.9%	Not performed	Not performed
Sheep	Ghosh et al. (2012)	Approx. 90,180	DHI (acc. to (Masuda et al. (2005))	90d post-injection DHI: 45%–50% decrease relative to baseline	Pfirrmann grading	Pfirrmann grading: 90d non-injected: Score 4–6 90,180d ChABC injected: Score 5–13
Sheep	Borem et al. (2021)	42, 119	DHI pre-injection vs. post injection	42d: −27% 119d: −30%	Pfirrmann grading	Pfirrmann grading: 17w ChABC injected: Score 1.25 ± 0.25 17w non-injected: Score 1 ± 0
Goats	Detiger et al. (2013)	84	DHI (acc.to Lü et al. (1997)) pre-injection vs. pre-autopsy	84d: −6%	MRI Index acc. to Sobajima et al. (2005); ChABC-injected vs. control	−22%
Goats	Hoogendoorn et al. (2008)	84, 126, 182	DHI (acc. to Lü et al. (1997)) pre-injection vs. pre-autopsy	84d: −12% 126d: −16% 182d: −20%	MRI Index acc. to Sobajima et al. (2005); ChABC-injected vs. control	84d: approx. −35% 126d: approx. −35% 182d: approx. −30%
Goats	(Study 1) (Hoogendoorn et al., 2007)	28, 56, 84, 126, 182	DHI (acc. to Lü et al. (1997)) pre-injection vs. pre-autopsy	28d: approx. −8% 56d: approx. −8% 84d: approx. −11% 126d: approx. −15% 182d: −13.8%	Qualitatively: acc. to Masuda et al. (2005); ChABC-injected vs. control Quantitatively: acc. to Sobajima et al. (2005) ChABC-injected vs. control	MRI Score (Masuda), average: 28d: approx. +0.4 56d: approx. +2 84d: approx. +0.5 126d: approx. +2.25 182d: n.a. MRI Index (Sobajima): 28d: approx. −22% 56d: approx. −40% 84d: approx. −15% 126d: approx. −45% 182d: n.a.
Goats	(Study 2) (Hoogendoorn et al., 2007)	84	DHI (acc. to Lü et al. (1997)) pre-injection vs. pre-autopsy	0.2 U/mL: approx. −12% 0.25 U/mL: approx. −12% 0.3 U/mL: approx. −17% 0.35 U/mL: approx. −24%	Qualitatively: acc. to Masuda et al. (2005); ChABC-injected vs. control Quantitatively: acc. to Sobajima et al. (2005) ChABC-injected vs. control	MRI Score (Masuda): 0.2 U/mL: approx. +0.5 0.25 U/mL: approx. +1.2 0.3 U/mL: approx. +1.5 0.35 U/mL: approx. +2.5 MRI Index (Sobajima): 0.2 U/mL: approx. −25% 0.25 U/mL: approx. −35% 0.3 U/mL: approx. −45% 0.35 U/mL: approx. −55%
Goats	Gullbrand et al. (2017)	84	DHI (acc. to Lü et al. (1997)), pre-injection vs. pre-autopsy	0.1 U: approx. −10% 1 U: approx. −20% 5 U: approx. −25%	Quantitative T2 and T1ρ relaxation times obtained from multi-echo and multi–spin-lock series, respectively; relaxation values measured within a manually segmented NP ROI (ImageJ) (acc. to Johannessen (Johannessen et al., 1990))	T2 0.1 U: approx. −15% 1 U: approx. −45% 5 U: approx. −55% T1rho 0.1 U: approx. −15% 1 U: approx. −40% 5 U: approx. −55% Pfirrmann grading: 0.1 U: approx. 2.5 1 U: 3.2 5 U: 3.8
Goats	Zhang et al. (2020)	84	Not performed	Not performed	Quantitative T2 and T1ρ mapping (multi-echo/multi–spin-lock sequences); NP relaxation times measured from circular ROI in ImageJ, acc. to Gullbrand et al. (2017), Nguyen et al. (2008), Gullbrand et al. (2016)	Progress of quantitative and qualitative MRI values not described Mod. Neg. correlation for T2 vs. TNFa- (r −0.57), IL-1ß- (r −0.57), IL-6- (r −0.46), ADAMTS-4- (r −0.63) expression Mod. Neg. correlation for T1rho vs. TNFa- (r −0.42), IL-1ß- (r −0.45), IL-6 (r −0.41), ADAMTS-4 (r −0.53)
Goats	Gullbrand, 2024	84	DHI (acc. to Gullbrand et al. (Gullbrand et al., 2017) and Martin et al. (Martin et al., 2017), pre-injection vs. pre-autopsy	By 70d: 2U: approx. −30.2% 5U: approx. −30%	Quantitative T2 mapping (CPMG sequence) in NP region by noise-corrected exponential fitting (per Meadows et al., 2020 (Meadows et al., 2020)); T1 mapping pre-/post-IV gadodiamide with %T1 reduction calculated as a diffusion metric (per Ashinsky et al. (Ashinsky et al., 2020))	84d: ∼15–20% T1 reduction in mild–moderate degeneration and ∼25–35% reduction in severe discs, ∼25–30% in healthy controls ∼36% reduction in NP T2 values in both 2U and 5U vs. controls
Goats	Peeters et al. (2015)	84, 168 (d0 ChABC injection; d84 hydrogel injection)	DHI (acc. to Lü et al. (1997)) pre-injection vs. pre-autopsy	84d: −6%	T2* relaxation time (calculated by fitting the echo time in- tensities using a linear-log least-squares method), compared to non-injected control	168d: −6%
Monkeys	Sugimura et al. (1996)	7, 42	DHI (acc. to Brandner)	7d: approx. −20% 42: approx. −35%	T2 signal intensity	Progress not described
Monkeys	Muramatsu et al. (2020)	1, 5, 9, 11, 13 17, 21, 26w	DHI pre-injection vs. post injection	Week1: 0.25U: −34.4% 10U: −40.5%	Not performed	Not performed
Bovine	Vernengo et al. (2023)	1-7d	DHI after dissection vs. day 7	7d: −0.06%	Not performed	Not performed

**TABLE 3:** Biomechanical findings in studies evaluating the degenerative potential of ChABC on large animal IVD.

Animal species	Study	Method	Findings
Goat	Detiger et al. (2013)	Lateral bending (LB) and axial rotation (AR) of motion segments	In LB and AR, NZ stiffness approx. - 31% and 27%, respectively. ROM +23% for LB and +32% in AR. No significant differences in flexion/extension
Goat	Gullbrand et al. (2017)	Motion segments subjected to cyclic tension/compression followed by creep	Significant increase in total ROM and NZ ROM for ChABC, and significant decrease in compressive modulus and NZ modulus, compared to intact control.
Goat	Gullbrand et al. (2024)	Creep indentation of facet cartilage using 0.1 N load for 15 min with biphasic creep model fitting (compressive modulus, tensile modulus, hydraulic permeability)	Marked increases in toe-region and linear-region stiffness in severe degeneration (+250–450%); no changes in strain parameters.
Goat	Paul et al. (2018b)	Changes in disk height recovery behavior were quantified using stretched-exponential fitting.	Increase of time required to reach 63% of the asymptotic value after the onset of the loading phase up to +50%
Sheep	Sasaki et al. (2001)	Intradiscal pressure measured by a catheter microtip pressure transducer	Post injection pressure decreased by 60%

**TABLE 4:** Microscopic findings in studies evaluating the degenerative potential of ChABC on large animal IVD.

Animal	Study	Follow-up	Method/Staining	Descriptive histology	Histologic grading method	Histologic grading results
Dogs	Fry et al. (1991)	5d, 21d	6 µm sections, HE, SOFG	Safranin-O Fast Green: All injected IVD showed ‘halo-zone’ (and analaogue eosinophilic zone in HE) HE: nuclear flattening, nuclear condensation, NP more eosinophilic and with fewer cells	Safranin-O Fast Green: 0 normal I perinuclear depletion in ventral annulus II perinuclear depletion with additional annular depletion III total annular and nuclear depletion	5d: 100% Grade I 21d: 72% Grade I; 28% Grade II
Sheep	Sasaki et al. (2001)	4w	HE, SOFG	IVD space narrowing Decreased SOFG in the NP and inner annulus	Not performed	Not performed
Sheep	Borem et al. (2021)	17w	7 µm sections, Alcain Blue, safranin-O, tartrazine, fast green	NP: regions of Degen IVDs exhibited a darkened and irregular matrix AF: regions showed disorganization CEP: integrity and uniformity were diminished, as evidenced by multiple protrusions into the subchondral bone along the endplate.	Thompson grading Rutges scale (Rutges et al., 2013): 0–2 normal 3–5 mild 6–8 moderate 9–11 severe 12–15 complete degeneration	Thompson grading: Uninjured: 1.0 ± 0.0 Injured: 4.0 ± 0.0 Rutges scale: Uninjured: 0.8 ± 0.8 Injured: 9.6 ± 0.4
Sheep	Ghosh et al. (2012)	84d	HE, Alcain Blue, neutral red	disruption of the structural integrity of the EP in some IVD most likely secondary to injection	Based on Sasaki et al. (2001): Grade I-IV	Mean score II-III
Goat	Hoogendoorn et al. (2008)	182d	7 µm sections, Alcain Blue	Day 182: some IVD presenting osteophytes, EP irregularities (destruction, subchondral osteolysis, presence of inflammatory cells, vascular ingrowth)	Adapted from Masuda et al. (2005)	Mean score: 84d: 4.2 126d: 3.8 182d: 3.5
Goat	(Study 1) (Hoogendoorn et al., 2007)	182d	7 µm sections, Alcain Blue, HE	NP ECM denser, loss of demarcation between NP/AF, AF lamellae changed to a more concave orientation compared to controls 84d, 182d: Some IVD with EP destruction, osteophyte formation	Adapted from Masuda et al. (2005)	Pearson correlation Histo Score vs. MRI Score = 0.68 Mean Score 28d: approx. 2.1 56d: approx. 2.2 84d: approx. 2.1 126d: approx. 3 182d: approx. 2.6
Goat	(Study 2) (Hoogendoorn et al., 2007)	84d	7 µm sections, Alcain Blue, HE	Similar to study 1	Adapted from Masuda et al. (2005)	Mean Score 0.2 U/mL: approx. 2.6 0.25 U/mL: approx. 4 0.3 U/mL: approx. 5 0.35 U/mL: approx. 5
oat	Gullbrand et al. (2017)	84d	Alcain Blue, Picrosirius red	In all doses: Endplate disruptions, characterized by apparent protrusions of NP material into the underlying bone 0.1U: in-folding of the AF lamellae, mild to moderate loss of proteoglycan staining in the nucleus pulposus, thinning of the cartilage endplate, fibrotic changes to the NP 1U and 5U: loss of proteoglycan in the NP, NP fibrosis, disorganization of the AF and loss of endplate structure	Adapted from Masuda et al. (2005) 0 (no) – 100 (most degeneration) on VAS	Mean Score 0.1U: approx. 30 1U: approx. 60 5U: approx. 75 Scores for 0.1 U were significantly lower regarding AF organization, AF/NP border, NP matrix
Goat	Mader et al. (2016)	Samples from Detiger et al. and Peeters et al. (Peeters et al., 2015; Detiger et al., 2013)	3–4 µm sections, HE, Alcain Blue, Masson Trichrome IHC for collagen 1 and 2	gradual increase of intensity from the NP towards the AF for collagen type I and decrease in intensity from the NP towards the AF for collagen type II and PG	Acc. to Hoogendoorn et al. (2007) 0 (no) – 6 (complete degeneration)	Data not provided
Goat	Zhang et al. (2020)	84d	Alcian Blue, Picrosirius red, HE	Not specified	Acc. to Gullbrand et al. (2017)	Data not provided
Goat	Gullbrand et al. (2024)	84d	Alcian Blue, HE, RGB Trichrome, SOFG	Overall, progressive NP proteoglycan loss, AF disorganization, and endplate involvement in severe cases. 2U: Initial proteoglycan loss in NP, Early AF disorganization, NP cellularity changes (cell clusters, early necrosis) 5U: Marked proteoglycan depletion in NP, Pronounced AF disorganization, Bony endplate defects resembling Schmorl’s nodes No significant differences in Bone/CEP sub-scores across groups	Acc. to JOR Spine/ORS Spine Section scoring system for large animals (Lee et al., 2021)	2U: Mean total histology score ∼12–13 (slightly ↑ vs. control), AF/NP structure ∼6, NP cellularity ∼7 (↑ vs. control), Bone/CEP unchanged (∼3). 5U: Mean total histology score ∼18–19 (marked ↑), AF/NP structure ∼9 (↑), NP cellularity ∼5–6, Bone/CEP unchanged (∼3).
Goat	Emanuel et al. (2018)	12 w	Alcian Blue, HE staining of midsaggital slices	Not specified	Acc. to Hoogendoorn et al. (2007) 0 (no) – 6 (complete degeneration)	Range 1.8–2.3
Goat	Paul et al. (2018b)	20d	3 µm sections, Alcain Blue, SOFG, HE	SOFG, Alcain Blue: NP staining is less homogeneous and lighter, oAF stained more intensively	Rutges scale (Rutges et al., 2013): 0–2 normal 3–5 mild 6–8 moderate 9–11 severe 12–15 complete degeneration	Range 4–7
Goat	Peeters et al. (2015)	84d, 168d (d0 ChABC injection; d84 hydrogel injection)	3 µm sections, Alcain Blue, HE	One IVD with fractured EP following injection	Acc. to Hoogendoorn et al. (2007)	168d Mean 2.57 ± 1.4
Monkeys	Sugimura et al. (1996)	7d, 42d	HE, toluidine blue, SOFG	Loss of staining of SOFG and toluidine blue NP fibrosis, decrease in NP cellularity	Not performed	Not performed
Monkeys	Muramatsu et al. (2020)	1, 4, 13, 26w	HE	NP decreased with degeneration/necrosis of NP cells, concentration of intercellular matrix. AF focal degeneration/necrosis, bulging of the AF toward the NP occurred. More severe at 10 U than 0.25 U	Not performed	Not performed
Bovine	Vernengo et al. (2023)	1-7d	SOFG	No significant changes in degeneration score at day 7	Modified acc. Lee et al. (2021)	p > 0.05

**TABLE 5:** ECM characterization in studies evaluating the degenerative potential of ChABC on large animal IVD.

Animal	Author	Follow-up (Days)	Method	PG evaluation	GAG evaluation
Dogs	Fry et al. (1991)	5, 21	SOFG staining	Depletion in ventral and dorsal anulus	Not evaluated
Dogs	Ono et al. (1998)	7–364	HPLC for molecular weight, acidity Carbazole assay (uronic acid) for PG quantity Gel filtration of PA-GAG for GAG-chain length assessment	PG quantity compared to control (no injection): 7d: −65% 14d: −84% 28d: approx. −30% 364d: −23% PG molecular weight change compared to control (no injection): 7d: decrease, 2 fractions (V0 and Kav 0.63–0.67) 28d: increase of V0, decrease of Kav 0.63–0.67 From 28d: recovery of V0 PG acidity compared to control (no injection): Lower compared to chymopapain group from week 8–52	7d: long and short chain 28d: only long chain
Goats	Hoogendoorn et al. (2008)	182	HPLC for total PG GAG/hydroxyproline ratio using a method by Burleigh et al. (1974)	Data not presented in the paper	GAG/Hyp ratio: Controls: 26:1–30:1 84d: 10:1 126d: 14:1 182d: 11:1
Goats	Hoogendoorn et al. (2007)	182	Alcian blue staining	Loss of staining	Loss of staining
Goats	Gullbrand et al. (2017)	84	Alcian blue staining	Loss of staining, dose-dependent	Loss of staining, dose-dependent
Goats	Gullbrand et al. (2024)	84	Alcian blue staining	Loss of staining, dose-dependent	Loss of staining, dose-dependent
Goats	Mader et al. (2016)	Samples from (Peeters et al. (2015), Detiger et al. (2013)	MCR-ALS of FTIR for total collagen, collagen I, collagen II, PG, elastin DMMB assay for GAG DMBA assay for Hyp	Two PG characteristics were found (PG1 and PG2) Higher PG concentration in NP and posterior AF	Correlated with PG, histological grading and MRI T2*
Goats	Emanuel et al. (2018)	168	MCR-ALS of FTIR for total collagen, collagen I, collagen II, PG, elastin DMMB assay for GAG DMBA assay for Hyp	PG content with strong correlation to GAG (DMMB) More PG in the nucleus, more collagen in oAF; sign. less in degenerated IVD in total and in NP only No sign. difference in PG entropy for degenerative and non-degenerative IVD; collagen entropy sign. higher in degenerated IVD	NP, DMMB/DMBA: no significant difference for GAG in degenerated and control IVD (365 vs. 290 ug/mg), Hyp (25.6 vs. 29.1 ug/mg), and ratio (18.1 vs. 11.1 g/g)
Goats	Paul et al. (2018b)	20	Spectrophotometric assessment (DMMB assay of papain digested samples), normalized to tissue dry weight SOFG, Alcian Blue staining	SOFG, Alcain Blue: NP staining is less homogeneous and lighter, oAF stained more intensively	GAG content significantly lower compared to PBS controls in NP: −16,5% iAF: +14,5% oAF: −28,8%
Goats	Peeters et al. (2015)	168	Spectrophotometric assessment (DMMB assay of papain digested samples), normalized to tissue dry weight DMBA assay for Hyp	Data not presented in the paper	−23% (Control: 376 μg/mg; ChABC: 290 μg/mg)
Sheep	Borem et al. (2021)	17w	GAG:hydroxyproline ratio method	Data not presented in the paper	GAG:HyPro ratio NP Region: Injured: −93.25 Uninjured: −83.48 AF Region: Uninjured: −99.15 Injured: −99.01
Monkeys	Sugimura et al. (1996)	42	HPLC for CS, KS, DS, HA	Data not presented in the paper	Content (nmol/disc) CS: −93% KS: +10%
Bovine	Vernengo et al. (2023)	7d	GAG content/wet tissue mass of iAF and oAF	Data not presented in the paper	iAF: ∼35% retention oAF: not statistically significant

#### 3.1.1 Chondroitinase properties

ChABC is a GAG degrading lyase produced by the bacteria *proteus* vulgaris. It selectively cleaves the PG’s side chains chondroitin-4-sulfate (chondroitin A), dermatan sulfate (chondroitin B) and chondroitin-6-sulfate (chondroitin C), and more slowly hyaluronic acid (Shaya et al., 2008; Ernst et al., 1995; Sugimura et al., 1996) at the 1,4-hexosaminidic bond via β-elimination (Prabhakar et al., 2005; Huang et al., 2003). The two types of chondroitinase ABC (type I: PvCSABCLyI, 997 amino acid residues; type II: PvCSABCLyII, 990 amino acid residues) exhibit different modes of action: while ChABC I is an endolytic enzyme, which degrades its substrates to tetrasaccharides and disaccharides, ChABC II is an exolytic enzyme with a different product distribution (Huang et al., 2003). Consistent with the endolytic character, the substrate-binding site in ChABC is a wide open cleft (Huang et al., 2003). ChABC does not exhibit protease activity and is not considered to be able to degrade proteoglycans (PG) completely (Fry et al., 1991). In this context, ChABC might be of value especially in the investigation of early degeneration, as the loss of PG marks the initial step in the vicious degenerative cycle (Paul et al., 2018a). In the large-animal IVD literature to date, no head-to-head comparison of type I versus type II ChABC in disc models has been reported, so any isoform-specific differences in degenerative profile remain unclear.

#### 3.1.2 Animal species, spinal sites and application

Chondroitinase induced IDD was investigated in dogs, goats, monkeys, bovine, and sheep (Table 1). Considering the age variation of the used animals, not all IVD can be considered ‘healthy’ (not degenerated) before the ‘induction’ of degeneration: Canine and goat IVD were up to 5 years old (Fry et al., 1991; Paul et al., 2018b; Gullbrand et al., 2017; Ono et al., 1998) while sheep IVD in the study of Sasaki et al. were up to 8 years old (Ghosh et al., 2012; Borem et al., 2021; Sasaki et al., 2001). Sugimura et al. used ‘mature’ rhesus monkeys (Sugimura et al., 1996), and Muramatsu et al. used Cynomolgus ages three to 5 years old (Muramatsu et al., 2020). Vernengo et al. used bovine IVDs less than 2 years old (Vernengo et al., 2023). Also, the follow-up time varied greatly within the different studies (one to 52 weeks in dogs (Lü et al., 1997; Ono et al., 1998), twelve to 26 weeks in goats (Detiger et al., 2013; Hoogendoorn et al., 2007), 26–28 weeks in monkeys (Sugimura et al., 1996; Muramatsu et al., 2020), one to 12 months in sheep (Ghosh et al., 2012; Borem et al., 2021; Sasaki et al., 2001), and one to 7 days in bovine (Vernengo et al., 2023)). Most of the authors performed *in vivo* injections under general anesthesia. The four *ex vivo* studies (Vernengo et al., 2023; Paul et al., 2018b; Castro et al., 2014; Paul et al., 2017) additionally applied ‘physiological’ mechanical loading to harvested IVD and performed the analysis within 22 days (Table 2).

The needle size has to be considered an important factor, as the injury secondary to puncture might already induce some degeneration. Although the size of lumbar IVD within certain species should be comparable, the injections were performed with needle of considerably different sizes (31–23 gauge in dogs (Lü et al., 1997; Ono et al., 1998), 29–21 gauge in sheep (Ghosh et al., 2012; Borem et al., 2021; Sasaki et al., 2001), 32–22 gauge in goats (Gullbrand et al., 2017; Paul et al., 2017), 31-gauge in monkeys (Sugimura et al., 1996; Muramatsu et al., 2020), and 29 gauge in bovine (Vernengo et al., 2023).

The volume of injected enzyme solution was comparable within particular species [0.05–0.1 mL in dogs (Fry et al., 1991), 0.1 to 0.2 in goats (Castro et al., 2014; Zhang et al., 2020), 0.01mL–0.02 mL in monkeys (Sugimura et al., 1996; Muramatsu et al., 2020), 0.2 mL in sheep (Borem et al., 2021; Sasaki et al., 2001), 0.1 mL in bovine (Vernengo et al., 2023)]. While overlapping, the amount of injected ChABC varied greatly across but also within different species: In canine IVD, 0.5–12.5 U ChABC were injected (Fry et al., 1991; Ono et al., 1998); in sheep, the injection consisted of 1–50 U ChABC (Borem et al., 2021; Sasaki et al., 2001). In goats 0.02–5 U ChABC (Hoogendoorn et al., 2007; Zhang et al., 2020) were applied. Sugimura et al. injected 4 U ChABC and Muramatsu et al. injected 0.25U and 10U in monkey IVD (Sugimura et al., 1996; Muramatsu et al., 2020). In bovine, Vernengo et al. injected 5 U/mL (Vernengo et al., 2023).

#### 3.1.3 Evaluation of the degenerative potential: disc height change

Most studies used x-ray imaging and some used calipers to quantify disc height loss (Table 2). In all animal species, a time dependency of this loss has been detected (Table 2). The use of different methods to assess disc height loss precludes a direct comparison of the published data (Table 2). Depending on the method, IVD height loss was quantified from −12% to −14% 1 week after injection (Ono et al., 1998; Takaishi et al., 1997), as −30% at 8 weeks and 0% 1 year after injection (regaining value of the normal group) (Ono et al., 1998). In sheep, height loss amounted to −6% to −15% after 1 week, −13% to −31% after 4 weeks (Sasaki et al., 2001), −27% after 6 weeks, −30% after 17 weeks (Borem et al., 2021), and was found to be as high as 50% at 12–15 weeks after injection (Ghosh et al., 2012). In goats, early IVD height loss has not been assessed (Table 2). Four and 8 weeks after injection, IVD height loss was shown to amount −8% (Hoogendoorn et al., 2007); at 12 weeks after injection, IVD height loss amounted −6% to −25% (Gullbrand et al., 2017; Detiger et al., 2013) while after 18 weeks it was around −15% (Hoogendoorn et al., 2007; Hoogendoorn et al., 2008) and after 26 weeks −14% to −20% (Hoogendoorn et al., 2007; Hoogendoorn et al., 2008). In monkeys, IVD height loss ranged from −20% to −66% after 1 week and −35% after 6 weeks (Sugimura et al., 1996; Muramatsu et al., 2020). A recovery of disc height in the longer term was described in dogs (Ono et al., 1998) and goats (Hoogendoorn et al., 2007). In bovine, 7 days post injection IVD height loss amounted −0.06% (Vernengo et al., 2023).

A dose-related effect has also been established in all animal models (Table 2). Higher doses generally resulted in a faster (Fry et al., 1991) and more extensive (Gullbrand et al., 2017; Sasaki et al., 2001; Hoogendoorn et al., 2007) height loss. Overdosing, on the other hand, seemed to diminish this effect (50U in sheep) (Sasaki et al., 2001).

#### 3.1.4 Evaluation of the degenerative potential: imaging

MR imaging was used to assess IVD degeneration quantitatively via relaxation times for T2 and T1rho signal and qualitatively using grading systems (Table 2). MRI has been performed most consistently in goat models but was also performed in sheep models. The absolute values of the scores are difficult to compare, considering their different composition of the included parameters.

Several authors (Peeters et al., 2015; Gullbrand et al., 2017; Paul et al., 2017; Zhang et al., 2020) detected a dose-dependent (Gullbrand et al., 2017; Paul et al., 2017) decrease of T2 signal intensity (which corresponds to the tissue water content) of −20% to −30% after 3 weeks (Paul et al., 2017), of −15% to −55% after 12 weeks (Gullbrand et al., 2017) and −6% after 24 weeks (Peeters et al., 2015) in ChABC-injected caprine IVD compared to controls. Similar observations have been made for the T1rho signal (a relaxation time parameter sensitive to low-frequency interactions between macromolecules, such as PG) (Gullbrand et al., 2017; Paul et al., 2017; Zhang et al., 2020). According to Paul et al., T1rho relaxation time correlates better than T2 with biomechanics, histology and matrix content (Paul et al., 2017). On the other hand, Zhang et al. found higher correlations of T2 with cytokine and catabolic enzyme expression levels compared to T1rho (Zhang et al., 2020).

More recently, Gullbrand et al. used quantitative T2 mapping in a cervical goat model injected with 2 or 5 U ChABC at C2–C3 and C4–C5 and observed ∼36% reductions in NP T2 values 12 weeks post injection compared to adjacent control levels, with NP T2 significantly correlating with histological degeneration scores (Gullbrand et al., 2024).

Using a quantitative MRI index designed as the product of computed nucleus pulposus area and average signal intensity by Borem et al. (2021), Sobajima et al. (2005), a dose-dependent decrease of −15% to −55% after 12 weeks was found by several authors (Borem et al., 2021; Detiger et al., 2013; Hoogendoorn et al., 2007; Hoogendoorn et al., 2008). Regarding IVD recovery, the correlation between MRI score, X-ray and relaxation time seems unclear, as Hoogendoorn et al. could not show a consistent long-term recovery of Sobajima’s score values at 26 weeks (Hoogendoorn et al., 2007; Hoogendoorn et al., 2008).

Qualitative grading was performed according to scores formulated by Pfirrmann et al. (Urrutia et al., 2016), DeCandido et al. (1988) and Masuda et al. (2005) Injected IVD consistently showed an increase of MRI grading values across the different scores used (Table 4). Once more, this was shown to happen in a dose-dependent manner (Hoogendoorn et al., 2007), while consistent recovery over time could not be established (Ono et al., 1998; Hoogendoorn et al., 2007).

Gullbrand et al. used a CT with an isotropic 20.5 μm resolution to detect a decrease of relative cortical bone volume and an increase of the adjacent trabecular bone volume in caprine IVD 12 weeks post injection (Gullbrand et al., 2017).

#### 3.1.5 Evaluation of the degenerative potential: biomechanical characterization

Four authors investigated biomechanical changes of large animal IVD following ChABC injection (Table 3).

In goats, mild IVD degeneration following ChABC-injection has been found to reduce neutral zone (NZ) stiffness, which is the range over which a spinal motion segment moves with minimal resistance (Smit et al., 2011) in lateral bending and axial rotation by approximately 30% (Gullbrand et al., 2017; Detiger et al., 2013). The same authors also showed an increase of total range of motion (ROM; +30 to +150%) compared to uninjected controls (Gullbrand et al., 2017) and lateral bending and axial rotation (approx. +20%) (Detiger et al., 2013). In the cervical spine, Gullbrand et al. (2024) reported that ChABC-induced degeneration led to progressive stiffening of the motion segment under axial compression: toe-region and linear-region moduli of severely degenerated discs increased by roughly 250%–470% compared to healthy and mildly degenerated discs, while creep strain and maximum compressive strain were not detectably altered.

Paul et al. investigated the recovery behavior of caprine IVD using stretched-exponential fits of the recovery of disc height following load release as obtained from displacement measurements in a bioreactor at day 20 and found the height recovery to take significantly longer in ChABC injected compared to PBS injected IVD (increase of time required to reach 63% of the asymptotic value after the onset of the loading phase up to +50%) (Paul et al., 2018b). Positively correlated with dosage, intradiscal pressure measured by a catheter microtip pressure transducer was shown to be decreased by up to 60% following ChABC injection into lumbar IVD in sheep (Sasaki et al., 2001). The authors negated a clear quantitative correlation between intradiscal pressure and IVD height index (Sasaki et al., 2001).

#### 3.1.6 Evaluation of the degenerative potential: microscopy

Seventeen authors performed histology in order to characterize degeneration and evaluate its severity following intradiscal ChABC administration (Table 4).

Alcian Blue (Ghosh et al., 2012; Peeters et al., 2015; Paul et al., 2018b; Gullbrand et al., 2017; Borem et al., 2021; Hoogendoorn et al., 2007; Paul et al., 2017; Zhang et al., 2020; Hoogendoorn et al., 2008; Emanuel et al., 2018; Mader et al., 2016) and Safranin-O staining (Vernengo et al., 2023; Sugimura et al., 1996; Fry et al., 1991; Paul et al., 2018a; Paul et al., 2018b; Borem et al., 2021; Sasaki et al., 2001; Paul et al., 2017) were used for GAG evaluation, Picrosirius red staining for collagen evaluation (Gullbrand et al., 2017; Zhang et al., 2020), and Hematoxylin-Eosin (H&E) staining was used for the evaluation of cellularity (Ghosh et al., 2012; Peeters et al., 2015; Sugimura et al., 1996; Fry et al., 1991; Paul et al., 2018b; Sasaki et al., 2001; Muramatsu et al., 2020; Hoogendoorn et al., 2007; Paul et al., 2017; Zhang et al., 2020; Emanuel et al., 2018; Mader et al., 2016).

From a descriptive perspective, IVD degeneration was characterized by a decrease in NP and perinuclear staining (SOFG) (Vernengo et al., 2023; Sugimura et al., 1996; Fry et al., 1991; Paul et al., 2018b; Gullbrand et al., 2017; Borem et al., 2021; Sasaki et al., 2001), nuclear flattening and condensation (Fry et al., 1991; Hoogendoorn et al., 2007), AF disorganization (Gullbrand et al., 2017; Borem et al., 2021; Muramatsu et al., 2020) and EP irregularities (Ghosh et al., 2012; Peeters et al., 2015; Gullbrand et al., 2017; Borem et al., 2021; Hoogendoorn et al., 2007; Hoogendoorn et al., 2008), decrease in concentration of intercellular matrix (Muramatsu et al., 2020). In goats, also osteophyte formation was observed (Hoogendoorn et al., 2007; Hoogendoorn et al., 2008).

Histological grading was performed by some of the authors in order to quantify the observed degeneration (Table 3. Score categories consisted of the evaluation of AF organization (Paul et al., 2018b; Gullbrand et al., 2017; Hoogendoorn et al., 2007; Paul et al., 2017; Zhang et al., 2020; Hoogendoorn et al., 2008; Emanuel et al., 2018; Mader et al., 2016), AF/NP border (Vernengo et al., 2023; Paul et al., 2018b; Gullbrand et al., 2017; Borem et al., 2021; Hoogendoorn et al., 2007; Paul et al., 2017; Zhang et al., 2020; Hoogendoorn et al., 2008; Emanuel et al., 2018; Mader et al., 2016), NP matrix (Vernengo et al., 2023; Paul et al., 2018b; Paul et al., 2017; Zhang et al., 2020; Hoogendoorn et al., 2008; Emanuel et al., 2018; Mader et al., 2016), NP cellularity (Vernengo et al., 2023; Paul et al., 2018b; Gullbrand et al., 2017; Borem et al., 2021; Paul et al., 2017; Zhang et al., 2020), and EP structure (Paul et al., 2018b; Gullbrand et al., 2017; Borem et al., 2021; Paul et al., 2017; Zhang et al., 2020).

Regarding these categories, differences between degenerated (ChABC injected) and non-degenerated (control, sham injection) discs were mainly observed for AF organization (Paul et al., 2018b; Gullbrand et al., 2017; Borem et al., 2021), AF/NP border (Paul et al., 2018b; Gullbrand et al., 2017; Borem et al., 2021) and NP matrix (Paul et al., 2018b; Gullbrand et al., 2017; Borem et al., 2021).

The variety of applied histological grading scores within the papers reviewed does not allow for a direct comparison. Nevertheless, the observed categorial histological score outcomes were related to a mild to moderate IVD degeneration (Peeters et al., 2015; Paul et al., 2018b; Borem et al., 2021; Hoogendoorn et al., 2007; Paul et al., 2017; Hoogendoorn et al., 2008; Emanuel et al., 2018), with only few authors reporting higher degeneration grades, especially following high dose ChABC application (Gullbrand et al., 2017; Hoogendoorn et al., 2007).

Similar to dose dependency, there seems to be a time-dependency of the induced degeneration. While Fry et al. noticed an increase in the score within 3 weeks in canine IVD (Fry et al., 1991), Hoogendoorn et al. described a decrease of score values after 12 weeks (Hoogendoorn et al., 2008), which indicates some recovery capacity.

Similar to their MRI findings, Zhang et al. showed a positive correlation of increased cytokine and catabolic enzyme mRNA expression levels and worse histological grading (IL-1ß, TNFa, ADAMTS-4 for NP and IL-1ß, ADAMTS-4 for AF) (Zhang et al., 2020).

#### 3.1.7 Evaluation of the degenerative potential: ECM characterization

Methods for the evaluation of PG and GAG content comprised of histology, HPLC, spectrophotometric assessment (DMMB assay of papain-digested samples) and infrared microscopy (Table 5).

Using a spectrophotometric assessment and DMMB assay in goats, Emanuel et al. did not detect a significant difference between degenerated and control IVDs at 168 days after administration of 0.25 U ChABC (Emanuel et al., 2018). On the contrary, Peeters et al. reported a reduction of GAG content of −23% for the 0.25 U ChABC-injected versus control IVD 168 days after injection (Peeters et al., 2015). However, the authors reported on large inter-animal differences (Peeters et al., 2015). Paul et al. were able to quantify GAG loss in degenerative caprine IVD 20 days after injection of 0.5 U/mL ChABC to an extent of 8.3% (Paul et al., 2018b). Interestingly, GAG reduction was found in the NP and outer AF, while there was an increase found in the inner AF (Paul et al., 2018b). Some authors showed a moderate (T2) and strong (T1rho) positive correlation (Paul et al., 2017) of GAG content normalized to tissue dry weight with MRI relaxation times (Paul et al., 2017; Mader et al., 2016) and histological grading (Mader et al., 2016).

GAG/hydroxyproline ratio of normal caprine IVD are comparable to healthy human samples (Mwale et al., 2004). Some authors used the GAG/Hyp ratio using DMMB and DMBA assays to verify the previously described increase in collagens and decrease in PG in mildly degenerated caprine IVD (Peeters et al., 2015; Hoogendoorn et al., 2008; Emanuel et al., 2018).

Borem et al. used GAG:HyPro ratio to determine the effect of ChABC injection on NP and AF ECM composition (Borem et al., 2021). The GAG:HyPro ratio in the NP region of Degen IVDs was 6.75 ± 4.34, compared to Uninjured IVDs 16.52 ± 4.63 as well as the vehicle IVD at 14.16 ± 4.83 (Borem et al., 2021). The GAG:hyPro ratio was significantly lower in the AF compared to the NP; in injured IVD the decreases was 0.99 ± 0.55, in uninjured it decreased by 0.85 ± 0.37, and decreased by 1.86 ± 1.23 in the vehicle IVD (Borem et al., 2021).

Vernengo et al. used tissue samples from each IVD region (30–50 mg) that were lyophilized and digested in 2 mL of 0.5 mg/mL proteinase K solution/10 mg of dry tissue and measured using 1,9-Dimethyl-methylene blue (Vernengo et al., 2023). GAG content/wet tissue mass of iAF and oAF was used to determine the effect of ChABC injection on ECM composition. In the iAF there was about a 35% retention of GAG content relative to day 0 (Vernengo et al., 2023).

Using a carbazole assay in order to evaluate the quantity of PG following ChABC-administration in canine IVD, Ono et al. were able to show a maximum PG depletion of 84% after 14 days (Ono et al., 1998). Some regeneration was recorded after almost 1 year (Ono et al., 1998). HPLC analysis revealed a distinct change of molecular weight (1 fraction in the control group, 2 fractions after 7 days and 1 fraction at the end of the follow up in the degenerative group) (Ono et al., 1998). Using gel filtration, the authors were also able to show that after 7 days, long and short GAG chain fragments were detectable, while after 28 days, there were only long chains (Ono et al., 1998).

More recently, Mader et al. demonstrated the potential of multivariate curve resolution-alternating least squares (MCR-ALS) of Fourier transform infrared (FTIR) microscopy in the evaluation of collagens, PG and elastin in degenerated IVDs in goats and human (Mader et al., 2016). Significant correlations of this multivariate analysis with histological grade, GAG content as assessed via DMMB, and MRI T2* measurements were observed (Mader et al., 2016). The authors concluded that this method might enable identification of novel components, modifications or degradation products which would not be possible using immunohistochemical analysis (Mader et al., 2016). Emanuel et al. evaluated Mader’s method in caprine IVD *in vivo* using ChABC and *ex vivo* using detrimental mechanical loading in a bioreactor and found the FTIR imaging to allow a more detailed investigation of early disc degeneration compared to other measures (Emanuel et al., 2018).

Gullbrand et al. also demonstrated clear dose-dependent ECM degradation in a cervical goat model, where higher ChABC doses led to proportionally greater loss of proteoglycan-rich matrix. Alcian blue and Safranin-O staining showed progressively reduced GAG content and diminished NP matrix integrity at 5 U compared to 2 U, with corresponding increases in AF matrix disruption. The work also demonstrated strong correlations between reduced NP proteoglycan staining and lower T2 relaxation times, indicating coupled structural and biochemical degeneration (Gullbrand et al., 2024).

#### 3.1.8 Evaluation of the degenerative potential: analysis of gene expression response

Evaluation of cytokine and enzyme expression levels was only performed in one of the studies reviewed. 84 days after injection of 0.1 U, 1 U or 5 U ChABC, Zhang et al. performed immunohistochemistry on caprine IVD (Zhang et al., 2020). Expression levels of TNFa and IL-1ß exhibited moderate correlation with the expression levels of the catabolic enzymes MMP-1, MMP-13, ADAMTS-4 as well as MRI T2 and T1rho relaxation time in the NP and AF (Zhang et al., 2020). For the NP, both cytokines were moderately correlated with the histological grading (Zhang et al., 2020). Among the catabolic enzymes, only ADAMTS-4 was significantly correlated with histological grading and MRI T2 and T1rho relaxation time in NP and AF (Zhang et al., 2020). However, IL-6 did not show a similar significant correlation with catabolic enzyme expression level or histological grading (Zhang et al., 2020). Higher doses (1 U and 5 U) resulted in a more consistent upregulation of cytokines and enzymes (Zhang et al., 2020).

#### 3.1.9 Complications and adverse effects

No complications, such as surgical complications, allergic reactions, or neurologic deficits have been reported by the studies reviewed (Sugimura et al., 1996; Fry et al., 1991; Borem et al., 2021; Hoogendoorn et al., 2008). Only Detiger et al. reported a single case of superficial wound infection after ChABC injection in goats (Detiger et al., 2013).

#### 3.1.10 Weighting of the degenerative potential

The expected outcomes of a GAG-degrading enzyme regarding IVD height loss, decrease of T2-signal intensity, decrease of biomechanical resistance, degradation of structural components of the NP, AF and EP and an increase of inflammatory cytokines and catabolic enzymes have been proven for ChABC. Taken together, these studies show that ChABC produces a tunable degeneration whose severity depends on species, dose, and follow-up time. In goats and sheep, low doses (≤0.25 U per disc) typically lead to approximately 5%–15% disc height loss with mild MRI and histological changes (Hoogendoorn et al., 2007; Hoogendoorn et al., 2008) whereas intermediate doses (0.5–5 U) yield ∼15–30% height loss accompanied by clear reductions in T2/T1ρ, GAG content, and neutral-zone stiffness (Vernengo et al., 2023; Gullbrand et al., 2017; Sasaki et al., 2001; Gullbrand et al., 2024). Very high doses (e.g., 50 U in sheep) paradoxically produced smaller height losses, though a mechanistic explanation for this was not explored (Sasaki et al., 2001). Gene-expression data Zhang et al. (2020) further indicate dose-dependent upregulation of inflammatory cytokines and catabolic enzymes. Across large-animal studies, ChABC demonstrated a favorable safety profile.

Comparative studies with ChABC are available related to chymopapain (Lü et al., 1997; Sugimura et al., 1996; Ono et al., 1998), papain (Vernengo et al., 2023), and collagenase (Vernengo et al., 2023). All three authors found the degenerative potential of ChABC to be lower compared to chymopapain: Lü et al. injected 2.5–5 U ChABC or 120 pKU chymopapain into 40 beagle IVD and found the latter to cause greater instability, greater height loss (up to ∼50–60% within 1–2 weeks), greater reductions in T2 signal, and greater PG depletion (Lü et al., 1997). Ono et al. showed less IVD space narrowing, less T2 signal intensity change and milder PG loss using 250 U/mL ChABC compared to 6 nKU/mL chymopapain in 50 µL injections into 140 beagle IVD (Ono et al., 1998). Also Sugimura et al., who injected 4 U ChABC or 200 pKU chymopapain into monkey IVD, found less IVD height loss, less T2-signal intensity change and less loss of staining (SOFG and toluidine blue) after ChABC injection (Sugimura et al., 1996). Vernengo et al. compared ChABC to papain and collagenase and discovered that ChABC best replicates early-stage degeneration more than papain and collagenase (Vernengo et al., 2023).

### 3.2 Chymopapain

Five studies investigated the degenerative potential of chymopapain in large animals, although it has been extensively used in human medicine for the treatment of disc hernia. One of these studies was designed to identify the regenerative potential of mesenchymal stem cells and platelet rich plasma in pigs (Chen et al., 2009).

#### 3.2.1 Chymopapain properties

Chymopapain originates from the same family of endopeptidases as papain; 58% of papain structure is identical to that of papain (Buttle, 2013). The prefix ‘chymo-’ of chymopapain stands for its higher ratio of milk-clotting to hemoglobin-digesting activity (Buttle, 2013). The enzyme consists of 218 amino acids organized in a single non-glycosylated polypeptide chain (Buttle, 2013). Besides the acceptance of hydrophobic residues in S2 and S3, chymopapain accepts a large range of side chains by other subsites (Buttle, 2013). Compared to papain, there are non-remarkable differences in substrate specificity as chymopapain reacts with 2,2′-dipyridyldisulfide at different rates and exhibits a second thiol group (Buttle, 2013).

Despite some well characterized clinical improvements secondary to intradiscal administration in humans (Hoogland et al., 2006; Nordby and Wright, 1994; Wardlaw, 2016), the degenerative potential of chymopapain has not been extensively investigated in large animals.

#### 3.2.2 Animal species, spinal sites and dosage

Among large animal species, intervertebral chymopapain injections were performed in pigs and dogs and monkeys (Table 6). Most injections were performed *in vivo* and animals were euthanized for further analysis after 7–182 days (Lü et al., 1997; Bradford et al., 1983) (Table 6). Using 31–20 G needles (Lü et al., 1997; Chen et al., 2009), 0.02–0.1 mL injections (Lü et al., 1997; Kudo et al., 1993) of 0.5–200 U chymopapain per IVD (Chen et al., 2009; Kudo et al., 1993) were used.

**TABLE 6:** Animal species, spinal sites and dosage in studies investigating the degenerative potential of chymopapain in large animals.

Animal	Sub-breed	Study	Region	Application	Adiitional features	Follow-up (Days)	Needle size (guage)	Volume (mL)	Amount
Pig	Miniature procine	Chen et al. (2009)	T13-L5	*Ex vivo*	Investigation of regenerative potential: MSC-GFP, PrP, MSC-GFP/PrP injection after 1 week	28	20	n.s.	n.s.
Pig	Miniature procine	Chen et al. (2009)	T13-L5	*In vivo*	Investigation of regenerative potential: MSC-GFP,PrP, injection after 1 week	54	20	n.s.	200U
Dogs	Mongrel	Kudo et al. (1993)	L1-5	*In vivo*	No	84	21	0.1	0.5 U; 5 U; 50 U
Dogs	Beagle	Lü et al. (1997)	L1-7	*In vivo*	No	7	31	0.02	120 pKU
Dogs	Mongrel	Bradford et al. (1983)	n.s.	*In vivo*	No	14, 84, 182	n.s.	0.1	1 mg

#### 3.2.3 Evaluation of the degenerative potential: disc height change

X ray imaging was used by several authors to investigate the disc height narrowing following chymopapain injection (Table 7). Various methods for DHI calculation were used. Performing radiographs at 7, 14, 28, 56 and 84 days after the injection, Kudo et al. showed maximum DHI decrease (up to −63% compared to pre-injection values) within 2 weeks followed by DHI recovery (Kudo et al., 1993). A clear dose dependency was not shown by the authors (Kudo et al., 1993). Lü et al. and Chen et al. used a comparable approach to estimate DHI in different species (Lü et al., 1997; Chen et al., 2009). 28 days after injection of 200 U chymopapain in porcine IVD, Chen et al. reported a DHI decrease of 27% (Chen et al., 2009). Lü et al. asserted a DHI decrease of −49% at 7 days after injecting 120 pKat into beagle IVD (Lü et al., 1997).

**TABLE 7:** Imaging findings in studies investigating the degenerative potential of chymopapain in large animals.

Animal	Study	Follow-up (days)	DH assessment	DH change	MRI signal assessment	Score change
Pig	Chen et al. (2009)	28, *In vivo*	X-ray for %DHI = (=verterbral body height/IVD height; relative to preoperative value	−27%	Not performed	Not performed
Dogs	Kudo et al. (1993)	7, 14, 28, 56, 84	X-ray for DHI= (=((anterior + posterior IVD height)/cranial EP width) x 100)	Maximum DHI reduction within 14d after injection (0.5U −63%; 5U −61%; 50U −58%	Not performed	Not performed
Dogs	Lü et al. (1997)	7	X-ray for %DHI (=verterbral body height/IVD height; relative to preoperative value)	More evident in the chymopapain conmpared to ChABC group; −48, 8%	Not performed	Not performed
Dogs	Bradford et al. (1983)	14, 84, 182	X-ray for DH assessment	14d: consistent narrowing; 84: beginning of reconstitution (no numbers provided)	Not performed	Not performed

#### 3.2.4 Evaluation of the degenerative potential: imaging

Besides x-ray imaging, two authors performed MR imaging to assess the degenerative potential of intradiscal chymopapain injections (Sugimura et al., 1996; Ono et al., 1998). At 1 week after chymopapain injection into canine IVD, T2-signal intensity began to decrease until signal disappearance after 8 weeks before recovering from week 13 on (Ono et al., 1998). Sugimura et al. did not explicate an observed T2 signal intensity decrease 6 weeks after intradiscal chymopapain injection in monkeys (Sugimura et al., 1996).

#### 3.2.5 Evaluation of the degenerative potential: biomechanical evaluation

Two studies included biomechanical testing of chymopapain injected IVD (Lü et al., 1997; Bradford et al., 1983). Lü et al. tested flexibility parameters (neutral zone stiffness and ROM) on canine motion segments without posterior elements in loading jigs (Lü et al., 1997). They found an increase of both parameters (spinal instability) following chymopapain injection in six degrees of freedom (F/E: NZ +340%, ROM +170%; LB: NZ +450%, ROM +155%; AR: NZ +400%; ROM +220%). Bradford et al. showed a 40%–50% decrease in axial stiffness and axial creep rate of chymopapain compared to uninjected canine IVD over 3 months (Bradford et al., 1983). Torsional stiffness decreased by approx. −30% at 3 weeks, but alongside torsional creep rate increased by approx. 30% at 3 months (Bradford et al., 1983).

#### 3.2.6 Evaluation of the degenerative potential: microscopy

All studies reviewed included histologic evaluation of chymopapain-induced IVD degeneration, however histologic grading was not performed. Most authors reported an extensive PG staining (Safranin-O) loss in the nucleus (Lü et al., 1997; Bradford et al., 1983). This effect seems to be dose dependent (Kudo et al., 1993). Lü et al. detected a loss of Safranin-O - staining particularly in the inner AF and NP 7 days after chymopapain injection (Lü et al., 1997), while Bradford et al. also reported on little to no Safranin-O- staining (PG) and decreased Fast-Green- staining (collagen) for the whole AF after 14 days (Bradford et al., 1983). According to the authors, this reflects the known effect of chymopapain diffusion out of the disc space (Bradford et al., 1983). In monkey IVD, Sugimura et al. reported on a loss of Toluidine Blue and Safranin-O staining (region not specified), fibrosis and decrease of number of chondrocytes in the NP regions 56 days after enzyme injection (Sugimura et al., 1996). The replacement of NP tissue with fibrocartilaginous tissue at 84 days (Kudo et al., 1993) and a similar appearance compared to controls at 182 days (Bradford et al., 1983) indicate some recovery after longer follow up periods.

#### 3.2.7 Evaluation of the degenerative potential: ECM characterization

Three authors investigated ECM changes by other means than histology (Ono et al., 1998; Chen et al., 2009). Using qPCR, Chen et al. found a strong decrease in gene expression levels of collagen II and aggrecan compared to uninjected controls in porcine IVD *in vivo* and *ex vivo* after up to 54 days (Chen et al., 2009). In canine IVD, Ono et al. used high-performance liquid chromatography (HPLC) to reveal a decrease in PG quantity (up to −85% after 7 days), which recovered over the experimental period (−23% after 1 year) (Ono et al., 1998). This was supported by the HPLC findings for GAG chain lengths (at 2 weeks shorter than long chains; at 4 weeks increase of long and decrease of short chains; at 8 weeks only long chains) (Ono et al., 1998). Sugimura et al. determined the content of GAG and side chains using HPLC and found a decrease in chondroitin-sulfate (CS; −60%), keratan-sulfate (KS; −50%), dermatan-sulfate (DS) and hyaluronic acid (HA; no numbers provided) in monkey IVD (Sugimura et al., 1996).

#### 3.2.8 Cytokines and catabolic enzymes

None of the studies reviewed contained information about the increase of cytokines or catabolic enzymes secondary to intradiscal chymopapain injection.

#### 3.2.9 Complications and adverse effects

Complications and adverse effects were not reported by any of the studies reviewed.

#### 3.2.10 Weighting of the degenerative potential

Comparative studies have been performed for chymopapain versus ChABC. In summary, chymopapain seems to have a stronger degenerative potential compared to ChABC:

In canine IVD, Ono et al. found T2 signal intensity change to be less in 250 U/mL ChABC compared to 6nKat/mL chymopapain injected IVD (Ono et al., 1998). Sugimura et al. found the same trend using 4 U ChABC or 200pKat chymopapain in monkey IVD, respectively (Sugimura et al., 1996). Regarding ECM degradation, Ono et al. showed changes in PG quantity and GAG chain length to be milder in ChABC injected IVD (Ono et al., 1998). Conversely, Sugimura et al. revealed a milder decrease of the side chains HA, CS and DS but a stronger decrease of KS 6 weeks after chymopapain injection (Sugimura et al., 1996). Histologic indicators of IVDD (loss of PG staining, NP fibrosis and cell loss) were more distinct following chymopapain compared to ChABC injection in monkeys (Sugimura et al., 1996). Lü et al. compared the potential of 120pKU chymopapain and 5U ChABC to induce IVDD in canine IVD at 1 week after *in vivo* intradiscal injection (Lü et al., 1997). Spinal segmental instability, IVD space narrowing (X-ray) and NP and AF PG destruction (SO-staining) were more distinct in the chymopapain compared to the ChABC group (Lü et al., 1997).

### 3.3 Collagenase

Literature search identified seven studies using collagenase to induce IVD degeneration in large animals. From these studies’ references no other articles were included. Most studies used a collagenase induced degeneration model to investigate treatment strategies, such as cross-linked hydrogels (Milani et al., 2012; Thor et al., 2017; Saunders et al., 2007) or gelatin (Growney Kalaf et al., 2014).

#### 3.3.1 Collagenase properties

Collagen is relatively resistant to proteolysis due to its tightly wound, semirigid triple helical structure (Ruangpanit et al., 2001). A group of enzymes capable of cleaving collagens type I, II and III was first described in 1962 (Ruangpanit et al., 2001). It is produced by C. histolyticum and consists of a mixture of collagenases and proteases. Since then, several collagenases have been purified and characterized, most of which belong to the family of matrix metalloproteinases (MMP-1, MMP-2, MMP-8, MMP-13, MMP-14, MMP-18, Cathepsin K) (Ruangpanit et al., 2001; Van Wart and Birkedal-Hansen, 1990). Other MMPs belong to the families of gelatinases (e.g., MMP-2), stromelysins (e.g., MMP-3), matrilysins (e.g., MMP-7), membrane-type MMP (MMP-14) and others that are not specified as collagenases (although exhibiting collagen-cleaving activities) (Wang W.-J. et al., 2015). Commercially available collagenases contain different preparations of partially purified subtypes I (col G gene of C. histolyticum) and II (col H gene of C. histolyticum) and proteolytic enzymes. Collagenases are classified as class I and class II, amongst others based on their relative activities toward collagen versus synthetic peptide substrates (Van Wart and Birkedal-Hansen, 1990). Furthermore, MMP-1 is aliased as collagenase-1, MMP-8 as collagenase-2, MMP-13 as collagenase-3 and MMP-18 as collagenase-4 (Wang W.-J. et al., 2015). The mode of action of collagenases involves an attack of the Yaa-Gly bonds in the repeating Gly-X-Y collagen sequence (Van Wart and Birkedal-Hansen, 1990).

#### 3.3.2 Animal species, spinal sites and application

Only bovine and goat tissue was investigated in studies about the degenerative potential of collagenase in large animal IVDs (Table 8). Animal’s age ranged from 7 months (Growney Kalaf et al., 2014) to 4 years (Antoniou et al., 2006). All studies used caudal IVD, except for Growney Kalaf et al. (2014), who investigated thoracolumbar IVD, Rustenburg et al. who investigated lumbar IVD (Rustenburg et al., 2020), and Vernengo et al. who investigated coccygeal IVD (Vernengo et al., 2023). All authors performed injections to apply the enzyme (Table 16). Additional procedures on the IVD consisted of mechanical loading within physiological range (Vernengo et al., 2023; Growney Kalaf et al., 2014), needle puncture (Growney Kalaf et al., 2014), and PBS injections (Milani et al., 2012; Saunders et al., 2007). Needle size ranged from 21 to 29-gauge and injection volume ranged from 0.04 to 1 mL. Most authors did not express the amount of injected enzyme consistently (e.g., in units) which prevents robust comparison (Table 9). In bovine IVD, Antoniou et al. injected 5 mg of 1370 U/mg in 40 µL solution per IVD (Antoniou et al., 2006) while Growney Kalaf et al. reported on an injection of 10–20 U in 1,000 µL solution (Growney Kalaf et al., 2014). Milani et al. injected 10 g/L (Milani et al., 2012), Thorpe et al. reported an injection of 2 mg/mL (Thor et al., 2017), and Vernengo et al. injected 0.5 U/mL (Vernengo et al., 2023). In goat IVD, Rustenburg et al. reported an injection of 1 mg/mL (Rustenburg et al., 2020). All analyses were performed within 2 days after enzyme administration.

**TABLE 8:** Animal species, spinal sites and application in studies evaluating the degenerative potential of collagenase on large animal IVD.

Animal	Sub-breed	Study	n IVD	Region	Specimen age	Application	Additional features	Follow-up (hours)	Needle size (guage)	Volume (mL)	Amount
Bovine	n.s.	Antoniou et al. (2006)	8	Caudal	2–4 years	Injection; *ex-vivo*	No	13–14	n.s	0.04	5 mg
Bovine	n.s.	Growney Kalaf et al. (2014)	n.s.	Thoracolumbar	28 weeks	Injection; *ex-vivo* for mechanical testin; incubation of NP specimens on glass coverslip for AFM	100-time needle insertion to stimulate annular tearing; MMP-3-group inhibition by TIMP; ‘light cyclic loading’: 30,000 cycles of 50–150N cyclic loading at 2 Hz	12, 24, 48	22	1	1 injection: 1% (20U); 0.5% (10U) collagenase; 0.0025% MMP-3 incubation: 0%, 0.1%, 0.5%, and 1.0% Collagenase-P (2.0 U/mg); 0% 0.00125%, 0.0025%, and 0.005% reconstituted MMP-3
Bovine	n.s.	Milani et al. (2012)	7	Caudal	18–30 months	Injection; *ex-vivo*	PBS injection after 18 h	18	n.s.	0.15	10 g/L
Bovine	n.s.	Saunders et al. (2007)	n.s.	Caudal	n.s.	Injection; *ex-vivo*	PBS injection after 18 h	18	n.s.	0.15	10 mg/mL
Bovine	n.s.	Thor et al. (2017)	10	Caudal	9–18 months	Injection; *ex-vivo*	No	2	21	0.1–0.2	2 mg/mL
Goat	milk goat	Rustenburg et al. (2020)	12	Lumbar	3–5 years	Injection; *ex-vivo*			29	0.05	1 mg/mL
Bovine	n.s.	Vernengo et al. (2023)	4	Coccygeal	<2y	Injection; *ex-vivo*	Physiological loading (0.02–0.2 MPa, 0.2 Hz, 2 h)	168	29	0.1	0.5 U/mL

**TABLE 9:** Imaging findings in studies evaluating the degenerative potential of collagenase on large animal IVD.

Animal species	Study	Follow-up (hours)	DH assessment	DH change	MRI signal assessment	Imaging findings
Bovine	Antoniou et al. (2006)	13–14	Not performed	1.5T; T1, T2, MT, ADC	1.5T; T1, T2, MT, ADC	T1: −20% compared to buffer injected control; T2: −25% compared to buffer injected control; MT and ADC without significant differences
Bovine	Saunders et al. (2007)	18	Relative IVD height reduction (pre- vs. pst loading cycles for mechanical testing)	Approx. −30%	Data not present in paper	Macroscopic evaluation: void formation in the NP region
Bovine	Thor et al. (2017)	2	Not performed	Not performed	Not performed	Macroscopic evaluation: void formation in 6/10 IVD following collagenase injection
Bovine	Vernengo et al. (2023)	168	DHI after dissection vs. day 7	7d: −1.2%	Not performed	Macroscopic evaluation: tissue voids at the center of the IVDs

#### 3.3.3 Evaluation of the degenerative potential: disc height change

Saunders et al. performed x-ray imaging to evaluate relative IVD height changes during a uniaxial mechanical testing period of 18 h and quantified the obtained height loss to approximately 30% compared to pre-injection levels (Saunders et al., 2007). Data for positive controls was not provided. Vernengo et al. also measured disc height daily for 7 days to calculate disc height change which resulted in a 1.2% decrease compared to day zero (Vernengo et al., 2023).

#### 3.3.4 Evaluation of the degenerative potential: Imaging

Macroscopic evaluation of collagenase treated IVD revealed void formation in the NP region (Vernengo et al., 2023; Thor et al., 2017; Saunders et al., 2007). Higher doses of collagenase seem to induce gross degradation, which according to Growney Kalaf et al. does not resemble the appearance of a naturally degenerated disc anymore (Growney Kalaf et al., 2014). From visualization, injection of 10 U collagenase most accurately mimicked IVD degeneration (pitted NP, lack of noticeable boundary between NP and AF) (Growney Kalaf et al., 2014). However, additional ‘light cyclic loading’ was applied (Growney Kalaf et al., 2014) (Table 9). Antoniou et al. performed MRI and found collagenase treatment without loading to significantly reduce T1 and T2 relaxation times at 13-h assessment (Antoniou et al., 2006). However, magnetization transfer (MT; number of protons in the semisolid pool relative to the total number of protons present; indicative for structural integrity) and the apparent diffusion coefficient (ADC; directly related to the proteoglycan content and inversely related to denaturated collagen) were not influenced (Antoniou et al., 2006).

#### 3.3.5 Evaluation of the degenerative potential: biomechanical characterization

Four studies performed biomechanical testing of collagenase injected bovine IVD (Table 10). Several biomechanical properties were shown to be reduced following the injection, such as elastic modulus (Milani et al., 2012; Saunders et al., 2007; Growney Kalaf et al., 2014), toughness (Milani et al., 2012), resilience (Milani et al., 2012), stiffness (Thor et al., 2017) and energy dissipation (Thor et al., 2017). Hysteresis, which expresses a water transport limitation effect (reswelling of NP tissue), was shown to be pronounced in collagenase injected IVD (Milani et al., 2012; Saunders et al., 2007).

**TABLE 10:** Biomechanical findings in studies evaluating the degenerative potential of collagenase on large animal IVD.

Animal species	Study	Method	Findings
Bovine	Growney Kalaf et al. (2014)	IVD specimen: Testing on the MTS 858 Mini Bionix II spinal loading machine and FlexTest GT System	Significant IVD height loss for collagenase (0.5% and 1%) compared to PBS injected IVD; degradation of the disk using collagenase resulted in a more rapid loss of disk height, as well as a greater displacement than MMP3 or PBS injection
Bovine	Growney Kalaf et al. (2014)	NP specimen: AFM for elastic modulus	Trend of decreasing elastic modulus for higher concentrations of MMP3 and collagenase; AFM testing was inconclusive as to which of the 2 higher collagenase concentrations would cause enough damage to the NP to mimic degeneration without inappropriate destruction to the disk
Bovine	Milani et al. (2012)	Static Compression tests using a Zwick-Rowll servo-hydraulic testing instrument and a purpose built load cell for compressive stress and strain	Toughness reduction (approx. 30%); resilience reduction; elasticity reduction; decreased maximum compressive stress and more pronounced hysteresis compared to uninjected controls: induced degeneration decreases the ability of the NP to reswell upon strain removal over the experimental period
Bovine	Saunders et al. (2007)	Uniaxial Compression tests using a Zwick-Roell servo-hydraulic testing instrument and a purpose-built load cell for compressive stress and strain	Pronounced hysteresis compared to microgel injected controls; reduction of elasticity modulus compared to microgel injected controls
Bovine	Thor et al. (2017)	Dynamic test (short period of walking) rig incorporating a hydraulic piston for stiffness, dissipated energy, strain	Stiffness: −88%; Strain: +80% Energy Dissipation: −180% compared to healthy IVD

#### 3.3.6 Evaluation of the degenerative potential: microscopy

Vernengo et al. was the only author to evaluate histology by staining the NP with SOFG (Vernengo et al., 2023). There was a complete loss of GAG staining in NP, a concentrated region observed in oAF for one sample, and high degeneration scores (Vernengo et al., 2023).

#### 3.3.7 Evaluation of the degenerative potential: ECM characterization

Antoniou et al. evaluated the ECM after intradiscal collagenase administration (Antoniou et al., 2006). While a colorimetric hydroxyproline assay suggested equal collagen content compared to buffer-injected IVDs, Western blotting revealed an increase of denatured collagen of 150% (Antoniou et al., 2006). The GAG content and GAG migration profile on the other hand were not influenced (Antoniou et al., 2006). GAG content was also evaluated by Vernengo et al. who found an increase in GAG content, however, the results were not significant enough in comparison to the controlled IVD (Vernengo et al., 2023).

#### 3.3.8 Evaluation of the degenerative potential: analysis of gene expression response

Only Vernengo et al. studied gene expression and discovered a mild downregulation in matrix markers (COL1, COL2, ACAN) and mild upregulation MMP3 (Vernengo et al., 2023).

#### 3.3.9 Complications and adverse effects

No information is available as there no preclinical or clinical studies were conducted on collagenase injections in the IVD.

#### 3.3.10 Weighting of the degenerative potential

Antoniou et al. compared eight bovine IVDs per group injected with 5 mg (1370 FALGPA U/mg) collagenase, 5 mg (>1500 U/mg) hyaluronidase or 5 mg (10,000 BAEE U/mg) trypsin using MRI and ECM characterization methods and found collagenase to exhibit the most detrimental effect on IVD (Antoniou et al., 2006). This is supported by atomic force microscopy results of Growney Kalaf et al., who noted that especially higher collagenase concentrations can cause inappropriate destruction to the disc (Growney Kalaf et al., 2014).

### 3.4 Papain

Nine studies evaluating the degenerative potential of intervertebral papain injection were included in the review. However, some used the degenerative model for other purposes (Bucher et al., 2013; Malonzo et al., 2015; Steffen et al., 2018) or used it to investigate a specific MRI method (Wang A. M. et al., 2015; Wan et al., 2020; Zuo et al., 2009). Hence, a comprehensive characterization of the degeneration induced by papain cannot be drawn from the existing literature.

#### 3.4.1 Papain properties

Papain belongs to the family of endopeptidases, which are proteolytic enzymes that break peptide bonds of nonterminal amino acids. Endopeptidases consist of 20 different families depending on a cysteine residue at the catalytic center (Rawlings and Barrett, 1994). Among them, the proteolytically active constituent in the latex of the tropical papaya fruit (Carica papaya) (Storer and Ménard, 2013), papain, is known to have a rather broad activity (Rawlings and Barrett, 1994; Storer and Ménard, 2013). In 1968, Drenth et al. first described the three-dimensional structure of this single-chain non-glycosylated polypeptide of 212 amino acids (Dre et al., 1968). The main effect of papain is the hydrolysis of its substrates, which consists of association, covalent binding (acetylation) and deacetylation (Storer and Ménard, 2013).

#### 3.4.2 Animal species, spinal sites and dosage

Almost all large animal studies reviewed used bovine caudal IVDs (Table 11). Needle size ranged from 22 to 29 gauge (Vernengo et al., 2023; Bucher et al., 2013; Malonzo et al., 2015; Chan et al., 2013), the injection volumes ranged from 0.05 to 0.15 mL (Wang A. M. et al., 2015; Wan et al., 2020; Chan et al., 2013) and the amount of injected enzyme ranged from 3 to 65 U/mL per IVD (Vernengo et al., 2023; Chan et al., 2013; Roberts et al., 2008). Some authors used 1 µM ebselen to inhibit the enzymatic activity of the enzyme (Malonzo et al., 2015). However, in several studies the needle sizes, volumes and doses of injected papain were not reported (Table 12).

**TABLE 11:** Animal species, spinal sites and application in studies evaluating papain induced IDD in large animals.

Animal species	Sub-breed	Study	Region	Needle size (guage)	Volume (mL)	Amount
Bovine	n.s.	Bucher et al. (2013)	Coccygeal	25	n.s.	60 U
Bovine	n.s.	Chan et al. (2013)	Caudal	25	0.08–0.14	3 U/mL, 15 U/mL
Bovine	n.s.	Malonzo et al. (2015)	Caudal	22	0.09	60 U/mL, 5.4 U/mL
Bovine	n.s.	Roberts et al. (2008)	Caudal	n.s.	0.07–0.1	20 mg/mL
Bovine	n.s.	Wang et al. (2015b)	Caudal	n.s.	0.05	1 mg
Bovine	n.s.	Wan et al. (2020)	Caudal	n.s.	0.05	1 mg
Bovine	n.s.	Zuo et al. (2009)	n.s.	n.s.	0.1	2 mg of 14U/mg
Dog	Beagle	Steffen et al. (2018)	Lumbrsacral	n.s.	n.s.	120 U/mL
Bovine	n.s.	Vernengo et al. (2023)	Coccygeal	29	0.1	65 U/mL

**TABLE 12:** Imaging findings in studies evaluating papain induced IDD in large animals.

Animal	Study	Follow-up (days)	DH assessment	DH change	MRI signal assessment	Score change
Bovine	Chan et al. (2013)	10	Not performed	Not performed	T2 weighted FSE	Narrowing of IVD space in all concentrations, shortening of T2 relaxation time in 60 U/mL and higher, injections of 30–150 U/mL lead to a degeneration resembing Watanabe’s Grade II-IV
Bovine	Roberts et al. (2008)	7, 14, 21	X-ray, caliper	Not reported	Not performed	Not performed
Bovine	Wang et al. (2015b)	5	Not performed	Not performed	DW-MRS, T2W-MRS, ADC mapping	Increase of macromolecules ADC and T2, decrease of macromolecule content; slight increase of water T2, content and ADC
Bovine	Wan et al. (2020)	n.s.	Not performed	Not performed	DW-MRS, T2W-MRS, CEST	Rapid increase of PG diffusivity, no significant PG and T2 change
Bovine	Zuo et al. (2009)	5	Not performed	Not performed	T2 weighted FSE, point-resolved spectroscopy (PRESS)	5d: significant decrease of N-acetyl peak height; correlated with GAG content
Bovine	Vernengo et al. (2023)	7	DHI after dissection vs. day 7	7d: −2.77%	Not performed	Not performed

#### 3.4.3 Evaluation of the degenerative potential: disc height change

Only Vernengo et al. evaluated disc height change. The authors measured the disc height change daily for 7 days which resulted in a 2.77% decrease compared to day zero (Vernengo et al., 2023).

#### 3.4.4 Evaluation of the degenerative potential: imaging

Imaging involving a variety of methods was performed in studies evaluating papain induced IDD (Table 12). Using diffusion weighted proton MR spectroscopy at 7 Tesla to detect resonance groups of the ECM macromolecules, Wang et al. showed an up to forty-fold increase of the macromolecule apparent diffusion coefficient and a slight increase of T2-signal 5 days after papain injection (Wang A. M. et al., 2015). Deriving the relative PG content from the N-acetyl resonance, Wang et al. and Zuo et al. found a slow and moderate decrease of PG (Wang A. M. et al., 2015; Zuo et al., 2009), which they discussed to be secondary to a gradual leak of PG fragments from the IVD (Wang A. M. et al., 2015). In line with this assumption, protein gel electrophoresis analysis revealed an increased amount of macromolecules with masses under 170 kDa in papain injected IVDs compared to sham injected control IVDs (Wang A. M. et al., 2015). At the same time, the water content as measured by T2 and apparent diffusion coefficient slightly increased (Wang A. M. et al., 2015) in papain injected IVDs compared to sham injected control IVDs. In future, beside MR spectroscopy chemical exchange saturation transfer (CEST) MR imaging seems promising to characterize the biochemical and physical properties of ECM macromolecules during early IVDD (Wan et al., 2020).

#### 3.4.5 Evaluation of the degenerative potential: biomechanical characterization

Biomechanical assessment of papain injected IVDs was only investigated by Chan et al. (2013). Using a servo-hydraulic testing machine, compressive stiffness and rotational stiffness were tested before and 10 days after papain injection (Chan et al., 2013). Compared to PBS injected controls, IVD dynamic compressive stiffness decreased by 10%–30% after 10 days following 60 U/mL and 150 U/mL injections at 0.5 Hz and 2 Hz, respectively (Chan et al., 2013). Rotational stiffness decreased by 60%–90% compared to controls 10 days after enzyme injection (Chan et al., 2013).

#### 3.4.6 Evaluation of the degenerative potential: microscopy

Histologic assessment was performed in bovine IVDs by four authors (Table 13). None of them used histologic grading. Cell loss and cavity formation was reported after high-dose (60–360 U/mL) enzyme injection in the NP region (Vernengo et al., 2023; Malonzo et al., 2015; Chan et al., 2013). Chan et al. found collagen fibers to be less organized in iAF and oAF compared to intact IVDs (Chan et al., 2013). Using Safranin-O-Fast-Green staining, Malonzo et al. and Vernengo et al. reported on a decrease of GAG in the AF (Vernengo et al., 2023; Malonzo et al., 2015). Roberts et al. found severe loss of metachromasia in toluidine blue stained sections (Roberts et al., 2008). Interestingly, although performing an *ex vivo* study in young animals’ tails (18–30 months), they found blood vessels in the oAF of enzyme injected and control IVD (Roberts et al., 2008).

**TABLE 13:** Microscopic findings in studies evaluating the degenerative potential of papain on large animal IVD.

Animal	Study	Follow-up (days)	Method/Staining	Descriptive histology
Bovine	Chan et al. (2013)	10	6 µm sections, HE, SOFG	HE: no cells in the NP of 60 and 150 U/mL injected IVD; loss of ECM in regions od AF and EP; parallel, but less organized collagen fibers in iAF and oAF
Bovine	Malonzo et al. (2015)	16	6 µm sections, SOFG	Decrease of GAG in AF; cavity formation in the NP
Bovine	Roberts et al. (2008)	7, 14, 21	4 µm sections, HE or toluidine blue	No cells in the digested NP region; severe loss of metachromasia; blood vessels in oAF of enzyme injected and control IVD
Bovine	Vernengo et al. (2023)	7	SOFG	Complete obliteration of the GAG staining across the sagittal cross-sectional area. produced statistically significant increases in degeneration grade compared to the PBS-inject controls at day 7 and day 0 intact samples (p < 0.05).

#### 3.4.7 Evaluation of the degenerative potential: ECM characterization

In bovines, GAG tissue content was determined by most authors with the DMMB assay (Table 14). Compared to PBS-injected IVD, GAG content was reported to be reduced by 67%–90% (Chan et al., 2013; Roberts et al., 2008). GAG/DNA ratio was found to be reduced by >90% compared to hydrogel injected IVD or PBS injected controls (Bucher et al., 2013; Malonzo et al., 2015). Bucher et al. reported a higher GAG loss in the outer AF compared to inner AF and NP (Bucher et al., 2013). Vernengo et al. evaluated GAG tissue content using the ratio of GAG content/wet tissue mass of iAF and oAF (Vernengo et al., 2023); in the iAF there was lesss then a 10% retention rate (Vernengo et al., 2023) and there was not a statistically significant change in the oAF (Vernengo et al., 2023).

**TABLE 14:** ECM characterization in studies evaluating papain induced IDD in large animals.

Animal	Author	Follow-up (days)	Method	PG evaluation	GAG evaluation
Bovine	Bucher et al. (2013)	21	GAG: DMMB	Not performed	GAG/DNA ratio iAF: approx. −90%; GAG/DNA ratio oAF: approx. −98%
Bovine	Chan et al. (2013)	10	GAG: DMMB	Not performed	Less than 10% GAG in oAF, iAF, NP of papain injected compared to uninjected IVD
Bovine	Malonzo et al. (2015)	16	GAG: DMMB	Not performed	GAG/DNA ratio in AF: approx. −90% compared to control
Bovine	Roberts et al. (2008)	21	GAG: DMMB	Not performed	GAG content reduced up to 66,7% compared to controls
Bovine	Zuo et al. (2009)	5	GAG: DMMB	Not performed	NP: significant correlation between N-acetyl/Lac + Lip and GAG content
Bovine	Vernengo et al. (2023)	7	GAG content/wet tissue mass of iAF and oAF	Not performed	iAF: <10% retention oAF: not statistically significant

Wang et al. further evaluated the macromolecule fragments generated from ECM degradation by using gel electrophoresis analysis 5 days after papain administration (Wang A. M. et al., 2015). They observed a dispersed distribution of the macromolecular masses under 170 kDa, indicating a wide variety of fragment sizes (Wang A. M. et al., 2015).

#### 3.4.8 Evaluation of the degenerative potential: analysis of gene expression response

Gene expression levels of cytokines and catabolic enzymes have only been investigated by Vernengo et al. (2023), Malonzo et al. (2015). Malonzo et al. detected an increase of gene expression levels for cytokines (IL-1ß, IL-6) and catabolic enzymes (ADAMTS-4, MMP-3, MMP-13) compared to controls 16 days after papain induced IVD degeneration (Malonzo et al., 2015). However, it should be noted, that the IVD were subject to static mechanical loading at 0.1 MPa from day 9–16, which may have contributed to the gene expression response (Malonzo et al., 2015). Vernengo et al. detected mild downregulation in matrix markers (COL2, ACAN) and a mild upregulation in ADAMTS5 and IL-1β (Vernengo et al., 2023).

#### 3.4.9 Complications, adverse effects

Adverse effects following papain injections regarding degenerative aspects have not been reported in studies investigating the degenerative impact of papain on large animal IVD. The studies’ follow up might have been long enough for most research purposes (Table 12), however, definitive conclusions towards possible negative effects cannot be drawn.

#### 3.4.10 Weighting of the degenerative potential

Only two studies compared the effect of papain and other enzymes on a large animal’s IVD: Roberts et al. compared the degenerative potential of papain and trypsin (Roberts et al., 2008). Performing dimethylmethylene blue assays 7, 14 and 21 days after enzyme injection into bovine IVD, they found the reduction of GAG content to be faster and more distinct in the papain compared to the trypsin group (Roberts et al., 2008). Hence, although trypsin might be preferable on a cost-basis, the authors suggested the use of papain in short interval studies (Roberts et al., 2008). Vernengo et al. compared the degenerative potential of papain, collagenase, and chondroitinase (Vernengo et al., 2023). The authors found that papain caused macroscopic tissue voids and the most significant loss in GAG content.

### 3.5 Trypsin

Ten studies characterizing the degenerative potential of trypsin were identified via literature search. From these studies references, another 2 articles (Hsu et al., 2013; Périé et al., 2006) were included. Some authors used trypsin in order to create a degenerative model for the investigation of the regenerative potential of different substances or treatments collagen-crosslinking via genipin (Hsu et al., 2013; Nikkhoo et al., 2016), platelet-rich- plasma (Nikkhoo et al., 2016), growth factors (short link N) (AlGarni et al., 2016; Mwale et al., 2008), mesenchymal stem cells (Mwale et al., 2008), traction treatment (Kuo et al., 2014).

#### 3.5.1 Trypsin properties

The serine protease trypsin, discovered in the 19^th^ century in pancreatic solutions, hydrolyzes peptide bonds C-terminal to the amino acid residues of lysine and arginine, so the natural substrate for the enzyme is generally any peptide that contains one of these two amino acids (Baird et al., 2006). This wide spectrum of substrates makes trypsin suitable for digestive as well as regulatory purposes (Baird et al., 2006). The catalytic center of trypsin is formed by serine, histidine and aspartate (Baird et al., 2006). The mechanism of action involves complex formation, acylation via hydroxyl group of serine and ionization, and deacylation via proton transfer (Hunkapiller et al., 1976).

#### 3.5.2 Animal species, spinal sites and application

Especially caudal bovine IVD were used for evaluation of the degenerative potential of trypsin (Table 15). In pigs, thoracic IVD were harvested. However, animal age ranged from <6 months to 4 years (Antoniou et al., 2006; Périé et al., 2006; Kuo et al., 2014). Trypsin administration was performed after IVD dissection by most authors. Except for one study, the enzyme was injected in the NP. Needle size was only recorded by three authors and ranged from 28.5 to 28 G (AlGarni et al., 2016; Mwale et al., 2008; Jim et al., 2011). The injection volume ranged from 0.04 mL to 0.5 mL (Mwale et al., 2008; Kuo et al., 2014) and the trypsin amount ranged from 50 μg to 5 mg (AlGarni et al., 2016; Mwale et al., 2008). Only Recuerda et al. incubated bovine IVD in a trypsin-containing solution instead of injecting the enzyme (Recuerda et al., 2012). Besides trypsin administration, some authors applied mechanical loading at ‘physiologic intensity’ (Table 15) (Hsu et al., 2013; Nikkhoo et al., 2016; Mwale et al., 2008; Recuerda et al., 2012). Roberts et al. stopped enzyme activity by serum injection 1 day after trypsin application (Roberts et al., 2008). Evaluation of degeneration was performed from immediately after enzyme application (Périé et al., 2006) to 28 days post-injection (AlGarni et al., 2016).

**TABLE 15:** Animal species, spinal sites and application in studies evaluating trypsin induced IDD in large animals.

Animal	Sub-breed	Study	n IVD	Region	Specimen age	Application	Additional features	Follow-up (days)	Needle size (guage)	Volume (mL)	Amount
Steers	n.s.	AlGarni et al. (2016)	6	Caudal	22–28 months	*Ex vivo*	One week preconditioning prior to enzyme injection	28	28, 5	0.04	50 µg
Bovine	n.s.	Antoniou et al. (2006)	8	Caudal	2–4 years	*Ex vivo*	No	13–14 h	n.s.	0.04	5 mg
Pigs	n.s.	Hsu et al. (2013)	8	Thoracic	<6 months	*Ex vivo*	Physiological loading: 16-h dynamic loading (0.2–0.8 MPa, 0.2 Hz), followed by an 8-h rest period (0.2 MPa)	7	n.s.	0.5	0.25%
Steers	n.s.	Jim et al. (2011)	8	Caudal	24–30 months	*Ex vivo*	No	4	28	N/A	2.5 µg/50 µL
Pigs	n.s.	Kuo et al. (2014)	16	Thoracic	6 months	*Ex vivo*	5 h fatigue loading on day 2; 24 h loading regime: 16 h dynamic loading 0.2–0.8 MPa, 0.2 Hz, 8 h static loading 0.2 MPa 16 h cyclic compression (50N-200N–50N at 1 Hz)	7	n.s.	0.5	0.25%
Bovine	n.s.	Mwale et al. (2008)	18	Caudal	2–3 years	*Ex vivo*	16 h cyclic compression (50N-300N–50N at 1 Hz)	16 h	n.s.	0.04	5 mg
Bovine	n.s.	Mwale et al. (2008)	18	Caudal	2–3 years	*Ex vivo*	No	16 h	n.s.	0.04	5 mg
Steers	n.s.	Mwale et al. (2014)	7	Caudal	24–30 months	*Ex vivo*	PBS injection 4 days after trypsin injection	18	28, 5	0.075	100 µg
Pigs	n.s.	Nikkhoo et al. (2016)	8	Thoracic	<6 months	*Ex vivo*	Physiological loading: 16 h dynamic loading (0.2–0.8 Mpa, 0.2 Hz), followed by an 8 h rest period (0.2 MPa)	7	n.s.	0.5	0.50%
Bovine	n.s.	Périé et al. (2006)	n.s.	Caudal	2–4 years	*Ex vivo*	No	0 h	n.s.	0.04	5 mg
Bovine	n.s.	Recuerda et al. (2012)	45	Caudal	n.s.	Trypsin incubation; *Ex vivo*	No	16 h, 18 h, 24 h	n.a.	n.s.	0.05 mg/mL, 0.1 mg/mL, 0.5 mg/mL
Bovine	n.s.	Roberts et al. (2008)	n.s.	Caudal	18–30 months	*Ex vivo*	Enzyme inhibition at day 1 via FBS injection	7, 14, 21	n.s.	0.07–0.1	0.1, 0.5, 1, 3 mg

#### 3.5.3 Evaluation of the degenerative potential: disc height change

Three authors used a caliper to assess IVD height course, with Kuo et al. reporting a four-fold increase in disc height loss compared to intact porcine IVD and Hsu et al. reporting on an IVD height loss of −30% compared to uninjected controls after 7 days (Hsu et al., 2013; Kuo et al., 2014).

#### 3.5.4 Evaluation of the degenerative potential: imaging

Roberts et al. described a cavity in the NP of bovine IVD following injections of trypsin, which was present for 21 days (Roberts et al., 2008). X-ray imaging after injecting a radio-opaque dye revealed that the cavity size increased with the amount of enzyme used (Roberts et al., 2008).

Many authors used MRI to characterize IVD degeneration following trypsin injection (Table 16). T1, T2 and magnetization transfer were shown to be not significantly affected by trypsin injection both with and without ‘physiologic loading’ (s. a.) across all studies (Antoniou et al., 2006; Périé et al., 2006; Mwale et al., 2008; Recuerda et al., 2012). While concordant to ECM findings (Périé et al., 2006), this seems to be in some contrast to biomechanical findings, indicating a higher sensitivity of biomechanical parameters compared to MRI features in response to structural damage (Périé et al., 2006).

**TABLE 16:** Imaging findings in studies evaluating trypsin induced IDD in large animals.

Animal species	Study	Follow-up (Days)	DH assessment	DH change	MRI signal assessment	Imgaing findings
Bovine	Antoniou et al. (2006)	13–14 h	Not performed	Not performed	1.5T; T1, T2, MT, ADC	T1, T2, MT: approx. Equal to buffer injected IVD; ADC increase in the lateral axis by 10%
Bovine	Mwale et al. (2008)	16 h, loading	Not performed	Not performed	1.5 T; T1, T2, MT, ADC, T1rho	NP and AF: T1 signal, T2 signal, T1rho signal, MTR and ADC: approx. equal to buffer injected IVD
Bovine	Mwale et al. (2008)	16 h, no loading	Not performed	Not performed	1.5 T; T1, T2, MT, ADC, T1rho	NP and AF: T1 signal, T2 signal, T1rho signal, MTR and ADC: approx. equal to buffer injected IVD
Bovine	Périé et al. (2006)	0 h	Not performed	Not performed	1.5T: T1, T2, MTR, TrD	T1, T2, MTR: reduced compared to buffer injected IVD, no significance; TRD increased compared to buffer injected IVD, no significance
Bovine	Recuerda et al. (2012)	6 h, 18 h, 24 h	Caliper	Not reported	3 T; T1, T2, MTR, ADC, FA	NP: ADC decrease (6 h: +2%; 18 h: −2%; 24 h: −6%), FA increase (6 h: >200%; 18 h: 12%; 24 h: >60%); AF: ADC decrease (6 h: +6%; 18 h: −3%; 24 h: −3%); FA increase (6 h: >50%; 18 h: >10%; 24 h: >30%); no significant changes in T1, T2, MTR
Bovine	Roberts et al. (2008)	7, 14, 21	Measured'	Data not provided	Not performed	Not performed
Pigs	Hsu et al. (2013)	7	Not specified	−30%	Not performed	Not performed
Pigs	Kuo et al. (2014)	7	Caliper	−0.43 mm vs. 0.1 mm in the control group	Not performed	Not performed

Regarding apparent diffusion coefficient controversial results were reported: while Recuerda et al. reported a gradual decrease following a 24 h incubation of bovine IVD in a trypsin solution (Recuerda et al., 2012), Antoniou et al. showed an ADC increase of 10% in the lateral axis 14 h after trypsin injection into bovine IVD (Antoniou et al., 2006). On the other hand, Mwale et al. did not show a significant difference in ADC of enzyme injected IVDs compared to buffer injected IVD after 16 h (Mwale et al., 2008). However, when combined with detrimental loading, MRI parameters indicated degenerative changes (Mwale et al., 2008). Overall, the follow up time in these studies might have been too short to detect significant changes by MRI following trypsin administration. Specialized imaging, like gagCEST imaging seems to be a promising mean for evaluating degeneration in the future (Saar et al., 2012).

#### 3.5.5 Evaluation of the degenerative potential: biomechanical characterization

Biomechanical characterization was performed by 5 authors (Table 17). Most of them evaluated compressive modulus and hydraulic permeability using confined compression tests. The compressive modulus, which expresses the ratio of the compressive stress applied to the IVD and the resulting IVD compression, was shown to be reduced by 50% (Recuerda et al., 2012), 70% (Mwale et al., 2008) and 90% (Périé et al., 2006) at 24 h, 14 h and 0 h after trypsin administration to bovine IVD, respectively. Mwale et al. (2008) and Périé et al. (2006) both injected 5 mg of trypsin per IVD, while Recuerda et al. used an incubation approach (Recuerda et al., 2012). Regarding hydraulic permeability, contradictory results have been reported. While in thoracic porcine IVD in a comparable experimental setting, hydraulic permeability decrease ranged from 20% to 25% 7 days after trypsin administration (Hsu et al., 2013; Nikkhoo et al., 2016), in caudal bovine IVD in a comparable experimental setting, hydraulic permeability increased by 20%–90% at the day of enzyme injection (Périé et al., 2006; Mwale et al., 2008), especially in NP regions (Recuerda et al., 2012). Besides the difference in investigated species (pigs vs. bovine), spinal sites of the animals (thoracic vs. caudal) and the length of follow up, the animals’ ages differed (porcine studies: <6months; bovine studies: 2–4 years). Further biomechanical findings indicated a decrease of IVD stiffness (−7 to −15%) and an increase of IVD bulging (Poisson’s ratio, +15%) in porcine IVD (Hsu et al., 2013; Nikkhoo et al., 2016). Bovine IVD showed a decrease of swelling pressure in NP regions (−85%) (Mwale et al., 2008).

**TABLE 17:** Biomechanical findings in studies evaluating the degenerative potential of trypsin on large animal IVD.

Animal species	Study	Method	Findings
Bovine	Antoniou et al. (2006)	Not performed	Not performed
Bovine	Mwale et al. (2008)	Confined compression testing in a compression chamber of NP and AF samples; Calculation of swelling pressure; computing of compressive modulus and hydraulic permeability	NP: swelling pressure: approx. −85%; compressive modulus: approx. −70%; hydraulic permeability: approx. +90% AF: approx. equal to buffer injected IVD
Bovine	Mwale et al. (2008)	Confined compression testing in a compression chamber of NP and AF samples; Calculation of swelling pressure; computing of compressive modulus and hydraulic permeability	NP: swelling pressure: approx. −85%; compressive modulus: approx. −70%; hydraulic permeability: approx. +90% AF: approx. equal to buffer injected IVD
Bovine	Périé et al. (2006)	Confined compression testing system: compressive modulus HA0 and hydraulic permeability k0	Compressive modulus: decrease > -90% compared to buffer injected IVD; hydraulic permeability: increase Approx. 20% compared to buffer injected IVD
Bovine	Recuerda et al. (2012)	Unconfined and confined compression tests, direct permeability measurement	NP: decrease of compressive modulus (6 h: >-50%; 18 h: >-35%; 24 h: >-50% of control group); increase of permeabilities (6 h: >30%; 18 h: >40%; 24 h > 60% compared to controls); AF: decrease of compressive modulus (6 h: >-15%; 18 h: >-30%; 24 h: >-60%); unclear change of permeabilities
Pigs	Hsu et al. (2013)	Linear biphasic model for aggregate modulus and hydraulic permeability; impulse testing for spring stiffness and damping coefficient	Aggregate modulus: approx. −20%; hydraulic permeability: approx. −20%; stiffness modulus: approx. −15%
Pigs	Nikkhoo et al. (2016)	Compression creep test, inverse FE model	Elastic modulus −7%; hydraulic permeability −25%; Poisson’s ratio (IVD bulging) +15%

#### 3.5.6 Evaluation of the degenerative potential: microscopy

Microscopical evaluation of trypsin induced IVD degeneration has been performed by seven authors (Table 18). Masson Trichrome, haematoxylin-eosin, Safranin-O and cell viability staining were performed. No study performed histological grading.

**TABLE 18:** Microscopic findings in studies evaluating the degenerative potential of papain on large animal IVD.

Animal species	Study	Follow-up (Days)	Method/Staining	Descriptive histology
Bovine	Roberts et al. (2008)	7, 14, 21	4 µm sections, H&E or Toluidine Blue	No cells within the nuclear region; healthy appearance of surrounding matrix; loss of metachromasia et al. l time points
Pigs	Hsu et al. (2013)	7	Masson trichrome	Slightly disorganized AF and slight condensation of the extracellular matrix in the NP region, comparable with Pfirrmann Grade I degeneration when Zhang’s grading would be applied
Pigs	Kuo et al. (2014)	7	SEM; Live-Dead: Calcein, Ethidium-homodimer	SEM: delamination of the AF; pore-occlusion by collagen mass; crimped collagen fibril bundles; micro-cracks in collagen fibers; Cell viability: 53% in NP, 22% in AF
Pigs	Nikkhoo et al. (2016)	7	Masson trichrome	Slightly disorganized AF and slightly condensed NP extracellular matrix
Steers	AlGarni et al. (2016)	28	5 µm sections, SOFG; cell viability using confocal	Decreased staining; >95% cell viability
Steers	Jim et al. (2011)	28	6 µm sections for cell viability via fluorescent dyes; 5 µm sections H&E and SOFG stained	No detrimental effect of trypsin injection to cell viability could be established; loss of SOFG staining after 14d
Steers	Mwale et al. (2014)	18	5 µm sections, HE, SOFG; confocal	SOFG: uniform loss of PG; PG depletion in NP region

Seven days after enzyme injection into porcine IVD, Nikkhoo et al. and Hsu et al. reported on slightly disorganized AF and slightly condensed NP extracellular matrix, corresponding to mild degeneration (Pfirrmann Grade I) (Hsu et al., 2013; Nikkhoo et al., 2016). Scanning electron microscopy revealed delamination of the AF, crimped collagen fibril bundles and micro-cracks in collagen fibers. Cell viability evaluation (Calcein/Ethidium Homodimer staining) revealed a decrease of ∼50% in the NP and ∼20% in the AF 1 week after trypsin injection, when an additional 5 h-fatigue loading (2 Hz, peak to peak: 190–590 N) was applied (Kuo et al., 2014). In 1 to 4- year old caudal bovine IVD, PG depletion and high cell viability (>95%) were reported after up to 4 weeks, also when an additional ‘physiologic loading’ was applied (AlGarni et al., 2016; Jim et al., 2011; Mwale et al., 2014). However, Roberts et al. reported on depletion in the nucleus but healthy appearance of surrounding ECM at one, two and 3 weeks after enzyme injection (Roberts et al., 2008).

#### 3.5.7 Evaluation of the degenerative potential: ECM characterization

Eight articles contained information about ECM degradation following trypsin administration (Table 19). Frequently, PG content was evaluated using a DMMB assay for tissue GAG and collagen content was evaluated using a colorimetric assay of tissue hydroxyproline content. Further evaluation methods comprised Western Blot and agarose gel electrophoresis (Antoniou et al., 2006; AlGarni et al., 2016; Jim et al., 2011; Mwale et al., 2014). Using a d0 control might be more appropriate for relating GAG loss than buffer injected IVD, as Jim et al. reported on a tissue GAG loss of approx. 25% after 4 days in PBS injected bovine IVD (Jim et al., 2011).

**TABLE 19:** ECM characterization in studies evaluating the degenerative potential of trypsin on large animal IVD.

Animal	Author	Follow-up (Days)	Method	Collagen evaluation	PG evaluation
Bovine	Antoniou et al. (2006)	13–14 h	DMMB assay of tissue for GAG; hdyroxyproline assay of tissue for collagen; agarose gel electrophoresis for PG fragments	HP assay for collagen: approx. equal to buffer injected IVD; WB: no significant effect on collagen denaturation	DMMB for GAG: approx. equal to buffer injected IVD; WB:change in the typical migration profile of aggrecan
Bovine	Mwale et al. (2008)	16 h, loading	DMMB assay of tissue for GAG; hdyroxyproline assay for collagen	NP: total collagen: approx. equal to buffer injected IVD; denaturated collagen: approx. +5% AF: approx. equal to buffer injected IVD	NP: approx. −30% AF: approx. equal to buffer injected IVD
Bovine	Mwale et al. (2008)	16 h	DMMB assay of tissue for GAG; hdyroxyproline assay for collagen	NP: total collagen: approx. equal to buffer injected IVD;denaturated collagen: approx. +3% AF: approx. equal to buffer injected IVD	NP: approx. equal to buffer injected IVD AF: approx. equal to buffer injected IVD
Bovine	Périé et al. (2006)	0 h	DMMB assay for tissue GAG; colorimetric assay of Hydroxyproline content for collagen	Total collagen: increase >40% compared to buffer injected IVD	DMMB: increase of GAG content approx. 14% compared to buffer injected IVD
Bovine	Roberts et al. (2008)	21	DMMB assay for tissue GAG	Not performed	GAG content lower than 10% CS/dry weight at 14d
Pigs	Nikkhoo et al. (2016)	7	DMMB assay	Not performed	NP: approx. −60%; iAF: approx. −60%; oAF approx. −10%
Steers	AlGarni et al. (2016)	28	DMMB assay of IVD tissue; Western Blot and densitometric analysis for collagen II and aggrecan G1	WB for collagen II: approx. −50%	DMMB: −50% compared to uninjected controls; WB for aggrecan: approx. −85%
Steers	Jim et al. (2011)	4, 8, 14	DMMB assay for tissue GAG; WB for chondroaherin and aggrecan	Not performed	DMMB: GAG loss 4d: −40%; 8d: 50%; 14d: −70% compared to d0 controls; WB d8: extensive loss of aggrecan (no numbers provided), 25% depletion of chondroadherin
Steers	Mwale et al. (2014)	18	DMMB assay for tissue GAG; agarose gel elelctrophoresis for PG; WB for aggrecan and collagen II	WB for collagen II: −40%	DMMB for GAG: −50% compared to uninjected controls; gel for PG: lower staining intensity; WB for aggrecan: −50%

In porcine IVD, GAG content has been shown to be reduced by −60% in NP and inner AF regions, while also outer AF regions showed a GAG reduction of −10% (Nikkhoo et al., 2016). The GAG reduction in AF regions has also been histologically observed (s. chapter 3.6.6). In bovines, DMMB assays showed a 40%–70% GAG reduction and western blots for aggrecan showed a reduction of −50% at 18 days and −85% at 28 days after enzyme injection (Antoniou et al., 2006; Jim et al., 2011; Mwale et al., 2014). The short term follow up studies in bovine IVD showed no difference or even slight increase in GAG content using DMMB in line with the MRI findings (Antoniou et al., 2006; Périé et al., 2006; Mwale et al., 2008) while Western blot and longer term follow up revealed a change in the typical migration profile of aggrecan (Antoniou et al., 2006) and GAG reduction (Roberts et al., 2008).

Total collagen content of trypsin injected bovine IVD was not reduced compared to buffer injected ones and western blots did not reveal a significant effect on collagen denaturation at 0–16 h after injection (Antoniou et al., 2006; Périé et al., 2006; Mwale et al., 2008). Additional ‘physiologic’ loading (50–300-50 N at 1 Hz for 16 h) led to an increase of denaturated collagen (Mwale et al., 2008) after 16 h. Contrary to these findings, longer term follow up studies in steer IVD showed a decrease of collagen II content of −40% to −50% without additional loading at 18 and 28 days, respectively (AlGarni et al., 2016; Mwale et al., 2014).

#### 3.5.8 Evaluation of the degenerative potential: analysis of gene expression response

To the best of our knowledge, the expression of inflammatory cytokine or catabolic enzymes in response to trypsin application have not been investigated.

#### 3.5.9 Complications and adverse effects

Complication or adverse effects resulting from trypsin intradiscal injections have not been reported in *ex vivo* studies. No studies report on trypsin injections in the IVD *in vivo*.

#### 3.5.10 Weighting of the degenerative potential

Differently from many other enzymes, trypsin has been shown to also affect AF regions when applied into the nucleus. In trypsin and papain injected IVD, cavity formation has been observed. On the other hand, MRI and histological findings in some studies suggest a rather mild degeneration. However, in these studies, the follow up might not be long enough to account for appropriate evaluation of degeneration or recovery. Antoniou et al. and Roberts et al. compared several enzymes regarding their degenerative potential in bovine IVD (Antoniou et al., 2006; Roberts et al., 2008). However, both study designs vary regarding the animals’ ages (18–30 years versus 2–4 years), the follow up length (21 days versus 14 h) and the amount of injected trypsin (0.1–2 mg versus 5 mg) (Table 3.6.2).

Antoniou et al. evaluated quantitative MRI and ECM after injecting collagenase, trypsin and hyaluronidase (Antoniou et al., 2006). Their results suggest a higher degenerative potential of trypsin followed by collagenase and then by hyaluronidase in bovine caudal IVD (Antoniou et al., 2006). Compared to papain, trypsin induced a slower GAG loss and a cavity formation in the NP only, whereas papain injection resulted in a cavity extension into AF regions after 3 weeks (Roberts et al., 2008).

## 4 Discussion

Low back pain associated with intervertebral disc (IVD) degeneration is a leading cause of disability and a major burden on healthcare systems worldwide. Addressing this condition requires translational research supported by physiologically relevant models of disc degeneration. Mechanical overload and needle puncture are among the most commonly used approaches in *ex vivo* and *in vivo* studies, offering straightforward and reproducible means to induce degenerative-like changes. However, these models carry significant limitations. Excessive mechanical force can result in severe disc disruption over the entire disc (Rivera Tapia et al., 2024; Zhou et al., 2024), while puncture injuries, particularly those made with large-gauge needles, may trigger acute injury responses (Michalek et al., 2010; Zhang et al., 2011; Hu et al., 2018) that fail to recapitulate the chronic, multifactorial progression of human degeneration. Additionally, both methods may cause immediate structural compromise, bypassing the biochemical and cellular cascades typically observed in clinical cases. These limitations highlight the need for alternative approaches. This paper focuses on proteolytic enzyme-based models as a means to more precisely simulate key features of disc degeneration, and explores their utility, limitations, and potential for advancing preclinical research.

**FIGURE 2:**
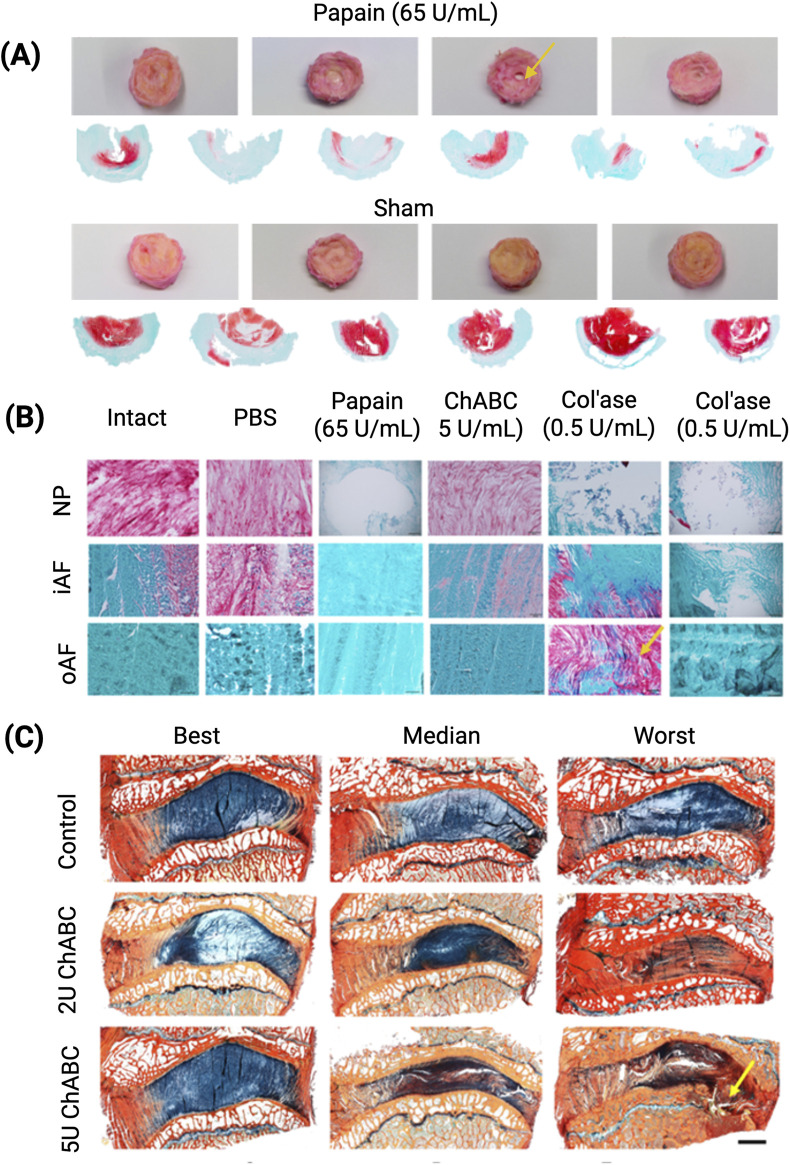
Comparative enzyme effects on bovine and goat IVDs. **(A)** Macroscopic cross-sections and X-rays show papain (65 U/mL) creates a large central cavity (yellow arrow), while Safranin-O/Fast-Green staining confirm result. **(B)** Safranin-O/fast-green histology (NP top, inner AF middle, outer AF bottom) reveals intense red proteoglycan staining in intact/PBS controls, near-total GAG loss with cavities after papain, patchy loss after collagenase II (col’ase), and mild pallor without voids after ChABC (5 U/mL). Yellow arrow for the col’ase group demonstrates a region of concentrated GAG staining observed in the oAF of one of the samples. Scale bars = 200 µm. **(C)** Goat cervical discs 12 weeks post-injection illustrate dose-tunable ChABC severity: 2 U yields subtle pallor, 5 U approaches papain-like matrix depletion. The yellow arrow denotes the location of an endplate resorption. Scale bars = 3 mm. In summary, papain reliably produces nucleotomy-like cavities for advanced-stage models, collagenase II yields intermediate voids, and ChABC—cavity-free at standard doses—spans mild-to-severe degeneration by adjusting units. **(A)** from Jansen et al. (2024) Gels 10:571 (Jansen et al., 2024), CC BY 4.0; **(B)** from Vernengo et al. (2023) Front. Bioeng. Biotechnol. 11:1178938 (Vernengo et al., 2023), CC BY 4.0; **(C)** from Gullbrand et al. (2024) Eur. Cells Mater. 47: 125 (Gullbrand et al., 2024), CC BY 4.0.

An overview of enzyme-induced intervertebral disc degeneration compared to healthy tissue is illustrated in Figure 2, highlighting some characteristic histological features and matrix changes produced by different proteolytic enzymes. Proteolytic enzyme models induce degeneration in a controlled and localized manner at the site of enzyme application, typically within the NP (Seguin et al., 2006; Antoniou et al., 2006). By degrading specific extracellular matrix components such as aggrecan, they mimic the matrix loss seen in natural degeneration (Sivan et al., 2014), leading to reduced NP hydration and elasticity. As degeneration progresses outward in these models, structural changes also emerge in the AF, resembling the fibrous-to-cartilaginous transition and lamellar disorganization observed in clinical pathology (Di Martino et al., 2005). Additionally, reductions in T2/T1ρ relaxation times generally parallel proteoglycan loss and increases in histological degeneration scores, and these changes often correspond with mechanical alterations such as increased range of motion or reduced neutral-zone stiffness (Lü et al., 1997; Sugimura et al., 1996; Gullbrand et al., 2017; Ono et al., 1998). While the strength of these correlations can differ across species and doses, the overall pattern demonstrates the cross-model coherence of enzyme-induced degeneration.

Nevertheless, several methodological factors require harmonization across studies. Enzyme dose, needle gauge, injection depth/speed, and the resulting spatial spread of the injectate directly influence the biochemical and structural pattern of degeneration and therefore limit reproducibility and comparison across studies. Standardization of these parameters will improve cross-study interpretation. As partial structural or compositional recovery has been observed at later time points (Ghosh et al., 2012), enzyme-induced changes are not always strictly progressive and follow-up duration must be carefully considered when interpreting severity.

Looking beyond their use as degenerative models, enzyme-based systems provide promising platforms for studying how the severity and biochemical nature of degeneration influence the success of interventions, including those involving mesenchymal stem cells (MSCs). MSCs are highly adaptable and can differentiate into multiple lineages, including disc-like cells capable of synthesizing extracellular matrix components characteristic of a healthy IVD (Peroglio et al., 2017; Christiani et al., 2021; Dai et al., 2021; Williams et al., 2021). In co-culture systems, MSCs have been shown to stimulate new matrix formation and adopt disc-specific phenotypes in response to biochemical cues from surrounding IVD cells (Naqvi and Buckley, 2016; Wuertz et al., 2008; Dalton et al., 2025). However, as degeneration progresses, the increasingly catabolic and inflammatory microenvironment, marked by elevated cytokines such as TNFα and IL-1β (Risbud and Shapiro, 2014; Zhang et al., 2020; Shnayder et al., 2023), can impair MSC function and limit their regenerative potential (Pourgholaminejad et al., 2016; Payne et al., 2025; Matta et al., 2021; Zhang et al., 2022). This underscores the need for preclinical models that reproduce not only the structural deterioration of the disc but also the biochemical signaling that governs cell behavior. Enzyme-induced degeneration models may meet this need by inducing matrix degradation, inflammatory signaling, and catabolic enzyme activity observed in human disc pathology. By allowing control over the extent of these degenerative features, enzyme models make it possible to develop and identify MSC-based or other regenerative strategies that are more likely to succeed.

Beyond serving as models of degeneration, enzyme systems can also be leveraged to dissect and optimize the regenerative process itself. For instance, chondroitinase ABC (ChABC) has been shown to upregulate COL2 expression in the inner annulus fibrosus (iAF) (Vernengo et al., 2023), a feature likewise observed in early-stage human degeneration (Cs-Szabo et al., 2002; Uei et al., 2006). Integrating enzyme-based models with global proteomic and genomic analyses (Xu et al., 2021; Wangler et al., 2021) at defined time points will enable deeper characterization of the molecular transitions that occur during both degeneration and repair. Such multidimensional profiling would help identify the key molecular changes that occur during degeneration, reveal which biological pathways are activated, and show when the disc is most responsive to treatment.

Looking ahead, a key challenge for the field will be bridging enzyme-based degeneration models with pain outcomes to establish a true translational link between bench and bedside. Without this connection, regenerative therapies risk advancing to clinical trials that restore tissue but fail to alleviate pain—the outcome that matters most to patients. Incorporating pain assessment into enzyme-induced models would directly connect the tunable biochemical and structural changes of chemonucleolysis with clinically relevant discogenic pain. A major opportunity lies in superimposing validated behavioral and functional readouts of disc pain onto enzyme-based degeneration in large animal models. In such studies, enzyme dose, injection depth, and spatial distribution could be calibrated not only to structural and molecular endpoints, as is standard now, but also to pain-related measures such as posture, mobility, weight-bearing asymmetry, activity monitoring, mechanical sensitivity, and spinal palpation responses (Lee et al., 2021). Likewise, wearable motion sensors can detect subtle gait asymmetries, and AI-driven facial recognition systems can identify early pain expressions with accuracy comparable to or exceeding that of expert observers (Figueirinhas et al., 2022; Feighelstein et al., 2025). Together, these technologies could allow researchers to pinpoint the enzyme dose or degeneration threshold that induces discomfort before overt lameness or distress appears.

While behavioral outcomes remain the most direct indicators of pain *in vivo*, molecular markers can also serve as valuable biochemical correlates for *ex vivo* models. Recent work in a rat chondroitinase ABC model identified pyruvate kinase M2 (PKM2) as a key mediator linking inflammation to pain hypersensitivity, with its inhibition reducing both molecular and behavioral pain indicators (Tripathi et al., 2025). These findings highlight the potential for enzyme-induced degeneration to activate pain-related signaling pathways. Continually identifying such markers through *in vivo* studies, particularly in large animal models, and evaluating their expression in *ex vivo* systems will make enzyme-based models increasingly predictive of nociceptive potential.

In summary, integrating enzyme-based large animal research with emerging behavioral, molecular, and AI-driven readouts can capture both the biological and experiential dimensions of disc degeneration. This integration will advance the field toward fully translational, ethically refined models that bridge the remaining gap between regenerative success in the laboratory and meaningful pain relief in the clinic.

## 5 Conclusion

This review demonstrates that proteolytic enzymes (ChABC, chymopapain, collagenase, and papain) can effectively induce intervertebral disc degeneration in large animal models, with each enzyme producing distinct degenerative patterns. ChABC is highlighted for modeling early-stage degeneration through selective glycosaminoglycan depletion, while enzymes such as papain generate more advanced matrix disruption. Across species, enzyme-induced degeneration consistently reproduces key features of human disc pathology, including disc height loss, altered biomechanics, and extracellular matrix changes, supporting their utility as platforms for studying disease mechanisms and testing regenerative therapies.

Looking ahead, continued refinement and standardization of enzyme dose and injection parameters will enhance reproducibility and comparability across studies. Integrating pain-related biomarkers into large-animal models offers a particularly important opportunity to strengthen their translational relevance and more directly connect structural degeneration with clinically meaningful outcomes.
